# Egocentric and allocentric representations in auditory cortex

**DOI:** 10.1371/journal.pbio.2001878

**Published:** 2017-06-15

**Authors:** Stephen M. Town, W. Owen Brimijoin, Jennifer K. Bizley

**Affiliations:** 1 Ear Institute, University College London, London, United Kingdom; 2 MRC/CSO Institute of Hearing Research – Scottish Section, Glasgow, United Kingdom; National Institute of Mental Health, United States of America

## Abstract

A key function of the brain is to provide a stable representation of an object’s location in the world. In hearing, sound azimuth and elevation are encoded by neurons throughout the auditory system, and auditory cortex is necessary for sound localization. However, the coordinate frame in which neurons represent sound space remains undefined: classical spatial receptive fields in head-fixed subjects can be explained either by sensitivity to sound source location relative to the head (egocentric) or relative to the world (allocentric encoding). This coordinate frame ambiguity can be resolved by studying freely moving subjects; here we recorded spatial receptive fields in the auditory cortex of freely moving ferrets. We found that most spatially tuned neurons represented sound source location relative to the head across changes in head position and direction. In addition, we also recorded a small number of neurons in which sound location was represented in a world-centered coordinate frame. We used measurements of spatial tuning across changes in head position and direction to explore the influence of sound source distance and speed of head movement on auditory cortical activity and spatial tuning. Modulation depth of spatial tuning increased with distance for egocentric but not allocentric units, whereas, for both populations, modulation was stronger at faster movement speeds. Our findings suggest that early auditory cortex primarily represents sound source location relative to ourselves but that a minority of cells can represent sound location in the world independent of our own position.

## Author summary

When we hear a sound, we can describe its location relative to ourselves (e.g., “the phone is on my right”) or relative to the world (e.g., “the phone is in the corner”). These descriptions of space are known as egocentric and allocentric, respectively, and illustrate the representation of sound location in reference frames defined either by the observer or the world. We know that neurons in the brain can represent the location of a sound source. However, previous experiments have been performed in static subjects, in which it’s not possible to tell whether spatial tuning reflects sensitivity to the position of the sound relative to the head or in the world. Here, we recorded neurons in the auditory cortex of freely moving ferrets and showed that most cells represent the position of a sound relative to the head, i.e., in an egocentric reference frame. We also recorded a smaller number of allocentric cells that were tuned to the position of a sound in the world, across the movement of the subject. By recording in freely moving animals, we were also able to investigate the neural encoding of sound source distance and the modulation of auditory processing by head movement speed.

## Introduction

A central role of the brain is to build a model of the world and objects within it that remains stable across changes in sensory input when we move. In hearing, this requires that an observer maintains the identification of an auditory object as they move through an environment. Movement is a critical aspect of sensing [1] that contributes to sound localization and other auditory behaviors [2–7]; however, the neural basis underpinning active hearing and how the brain constructs world-centered sound location remains unknown.

For a moving observer, it is possible to represent sound location either relative to oneself (egocentric representation) or relative to the world through which one moves (allocentric representation). Allocentric representations provide a consistent report of object location across movement of an observer [8], as well as a common reference frame for mapping information across several observers or multiple sensory systems [9,10]. Despite the computational value and perceptual relevance of allocentric representations to hearing, studies of auditory processing have only recently considered the coordinate frames in which sound location is represented [11–13]. Both electroencephalography (EEG) and modelling studies hint that sound location might be represented in cortex in both head-centered and head-independent spaces. However, EEG has not yet revealed the precise location of these representations and cannot determine how individual neurons in tonotopic auditory cortex define space.

In static subjects, auditory cortical neurons encode sound azimuth and elevation [14–18], and localization of sound sources requires an intact auditory cortex [19–21]. However, in static subjects with a fixed head position, neural tuning to sound location is ambiguous, as the head and world coordinate frames are fixed in alignment, and so allocentric and egocentric sound location are always equivalent. While it has been largely assumed that cortical neurons represent sound location relative to the head, the spatial coordinate frame in which location is encoded remains to be demonstrated. Furthermore, though the acoustic cues to sound localization are explicitly head-centered, information about head direction, which is necessary to form a world-centered representation, is present at early levels of the ascending auditory system [22]. Thus, it may be possible for neurons in the auditory system to represent space in an allocentric, world-centered coordinate frame that would preserve sound location across changes in head position and direction.

Here, we resolve the coordinate frame ambiguity of spatial tuning in auditory cortex by recording from neurons in freely moving ferrets. In moving conditions, the head and world coordinate frames are no longer fixed in alignment, so we can determine in which coordinate frame a given cell is most sensitive to sound source location. Our approach reveals head-centered and world-centered units, suggesting that egocentric and allocentric representations coexist in auditory cortex. We also explore the impact of distance from a sound source and the speed of a subject’s movement on spatial tuning in auditory cortex.

## Results

**Fig 1:**
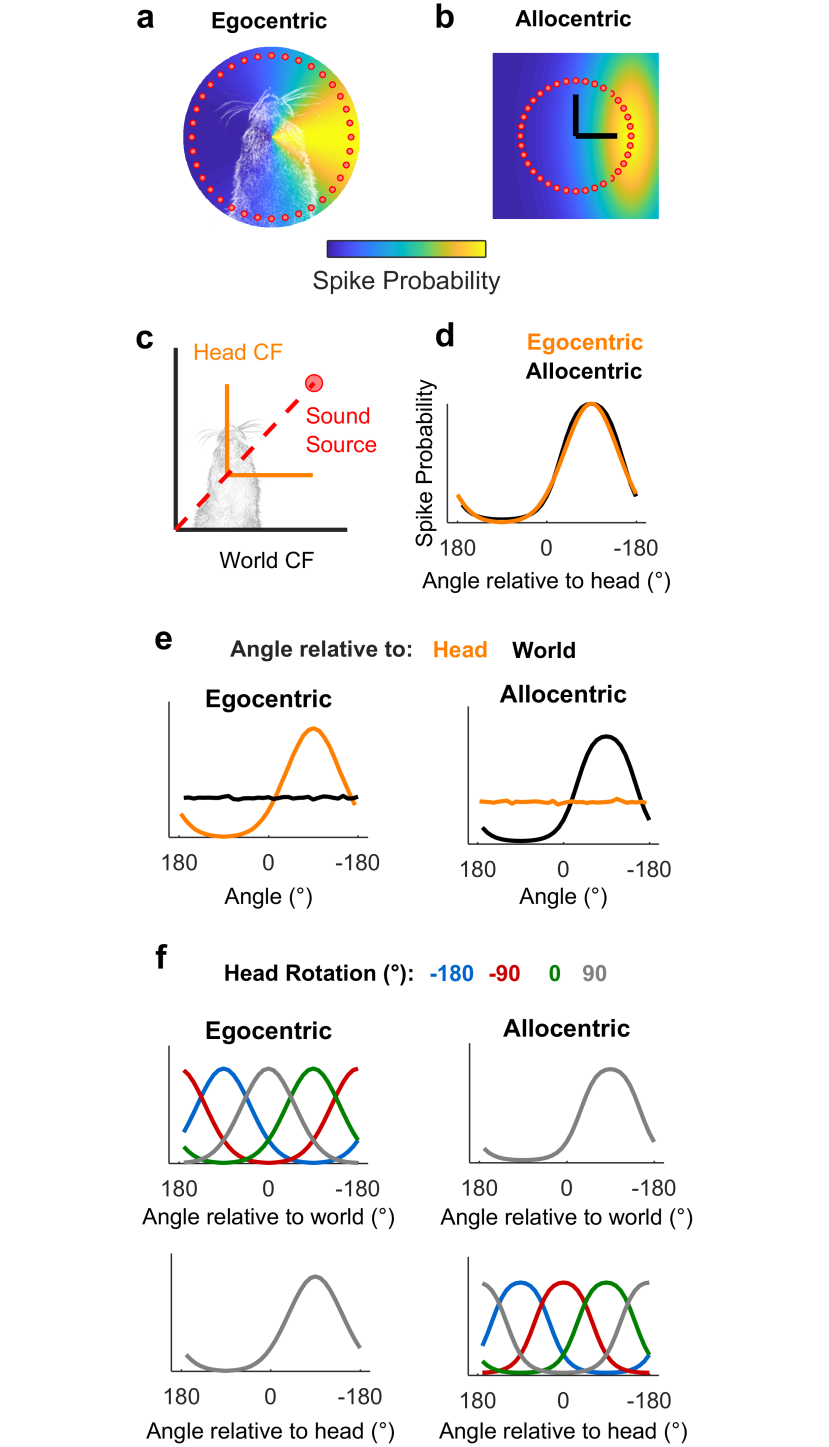
Simulated receptive fields show that observer movement resolves coordinate frame ambiguity. **a-b**: Simulated neurons with receptive fields tuned to sound location relative to the head (a, Egocentric) or in the world (b, Allocentric). Circles show hypothetical sound sources in a classical speaker ring; black lines indicate axes and origin of the simulated world. **c**: Schematic of world and head coordinate frames (CFs). **d**: Sound-evoked tuning curves according to allocentric and egocentric hypotheses when head and world coordinate frames were aligned. **e-f**: Predictions of allocentric and egocentric hypotheses showing mean spike probability averaged across uniform distributions of head rotation and position (e) and at specific head directions (f). Data available at https://doi.org/10.6084/m9.figshare.4955210.v1.

We hypothesized that measuring spatial tuning in moving subjects would allow us to distinguish between egocentric (head-centered) and allocentric (world-centered) representations of sound location (Fig 1). To formalize this theory and develop quantitative predictions about the effects of observer movement on spatial tuning, we first simulated egocentric and allocentric neurons that were tuned to sound locations defined relative to the head (Fig 1a) and world (independent of the subject), respectively (Fig 1b). We simulated allocentric and egocentric units using parameters fitted to produce identical spatial receptive fields when tested in the classical condition in which the head is in a fixed location at the center of a speaker ring (Fig 1c and 1d), illustrating coordinate frame ambiguity. Our simulation also confirmed that when the observer moved freely with a uniform distribution of head directions, spatial tuning should only be apparent in the coordinate frame relevant for neural output (Fig 1e). Additionally, changes in head direction produced systematic shifts in tuning curves in the coordinate frame that were irrelevant for neural output, while tuning in the relevant coordinate frame was invariant across head direction (Fig 1f). We subsequently demonstrated that tuning curves of many shapes and preferred locations can theoretically be explained by spatial receptive fields based within an allocentric coordinate frame (S1 Fig). With simulations providing a foundation, we then made recordings in freely moving animals to determine whether the spatial tuning of auditory cortical neurons followed egocentric or allocentric predictions.

**Fig 2:**
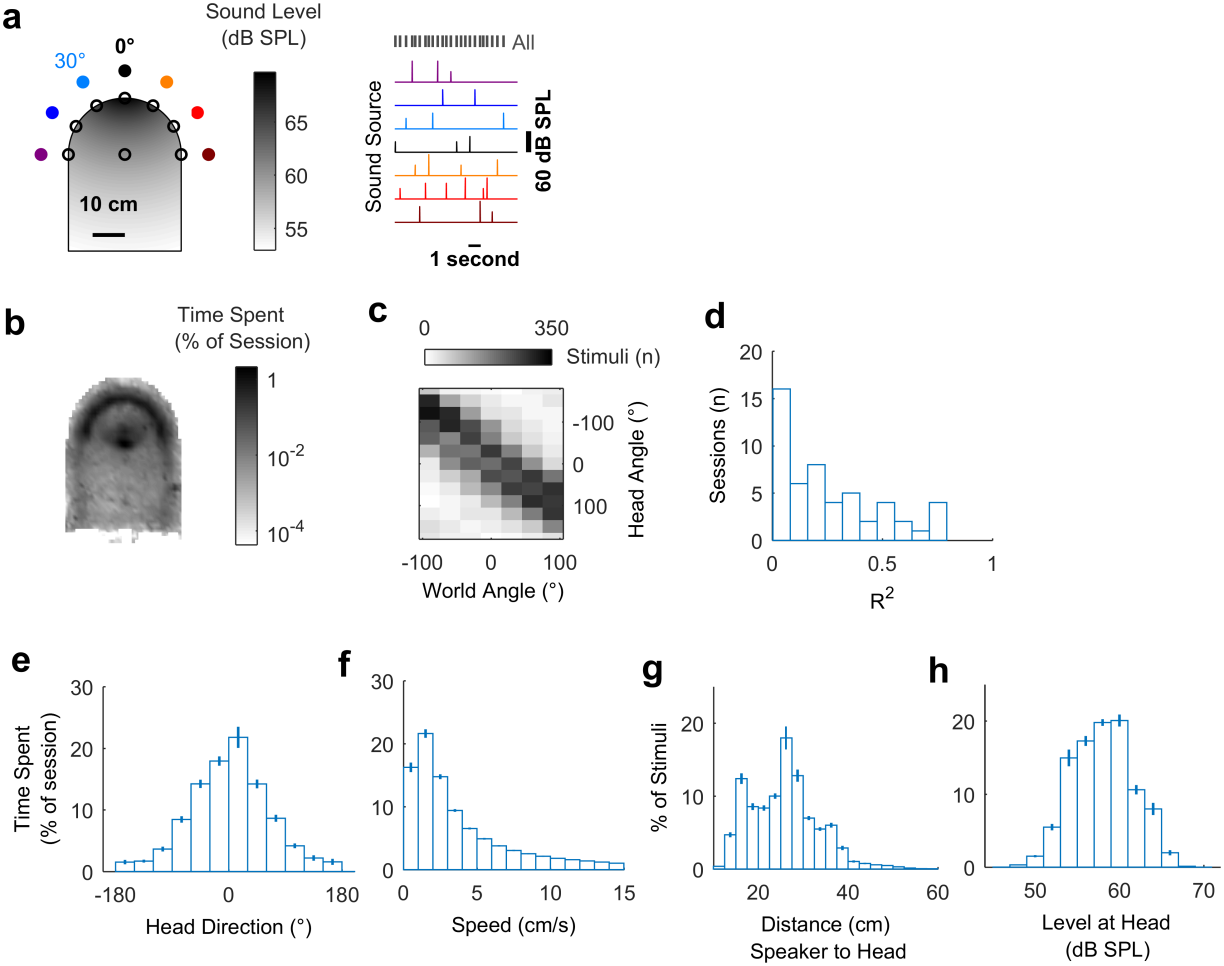
Experimental design and exploratory behavior in a sound field. **a**: Arena with speakers (filled circles) and water ports (unfilled circles). Shading indicates the sound field generated by a click from the speaker at 0°, calibrated to be 60 dB SPL at the center of the chamber. Stimuli were presented with a pseudorandom interval and order across speakers. **b**: Mean proportion of time in each recording session spent within the arena. **c**: Stimulus angles relative to the head and world for one session that was representative of behavior in all sessions (*n* = 57). **d**: Correlation coefficients (R^2^) between sound angles in head and world coordinate frames across all behavioral sessions. **e-h**: Distributions of head direction, head speed, distance between head and sound source, and the sound level at the animal’s head during behavior. Bars indicate mean ± SEM across sessions. Data available at https://doi.org/10.6084/m9.figshare.4955258.v1.

To measure spatial tuning in moving subjects, we implanted ferrets (*n* = 5) with multichannel tungsten electrode arrays, allowing the recording of single and multiunit activity during behavior. During neural recording, each ferret was placed in an arena, which the animal explored for water rewards while the surrounding speakers played click sounds (Fig 2a). To measure the animal’s head position, direction, and speed in the world during exploration (Fig 2b–2f), we tracked light-emitting diodes (LEDs) placed on the midline of the head (S1 Video). During exploration, click sounds were presented from speakers arranged at 30° intervals between ±90° relative to the arena center, with speaker order and inter-click interval (250–500 ms) varied pseudo-randomly. We also roved the level of clicks between 54 decibel sound pressure level (dB SPL) and 60 dB SPL such that absolute sound level varied both as a function of sound source level and distance between head and speaker, to reduce cues about sound location provided by absolute sound level (Fig 2g and 2h). Clicks were used as they provided instantaneous energy and thus ensured minimal movement of the animal during stimulus presentation (S3 Fig). The locations (speaker angle) from which the clicks originated were used alone to estimate **allocentric** receptive fields and were used in conjunction with the animal’s head direction and position to measure **egocentric** spatial receptive fields.

We observed that animals moved throughout the arena to collect water (Fig 2b) and used a range of head directions during exploration (Fig 2e). In contrast to our initial simulations, the distribution of the animal’s head direction was notably non-uniform, leading to correlations between sound source angle relative to the head and the world (e.g., Fig 2c; mean ± SEM R^2^ = 0.247 ± 0.0311). This correlation between sound source angles resulted because the animal preferred to orient towards the front of the arena (0°) and thus sounds that were to the right of the animal were more often on the right of the arena than would result from random behavior. The preference of the animal was likely a consequence of the shape of the arena and the location of the water spouts within it. Although the correlation between sound source locations relative to the head and within the world was relatively small, we sought to determine how the animal’s head direction preference affected our experimental predictions.

**Fig 3:**
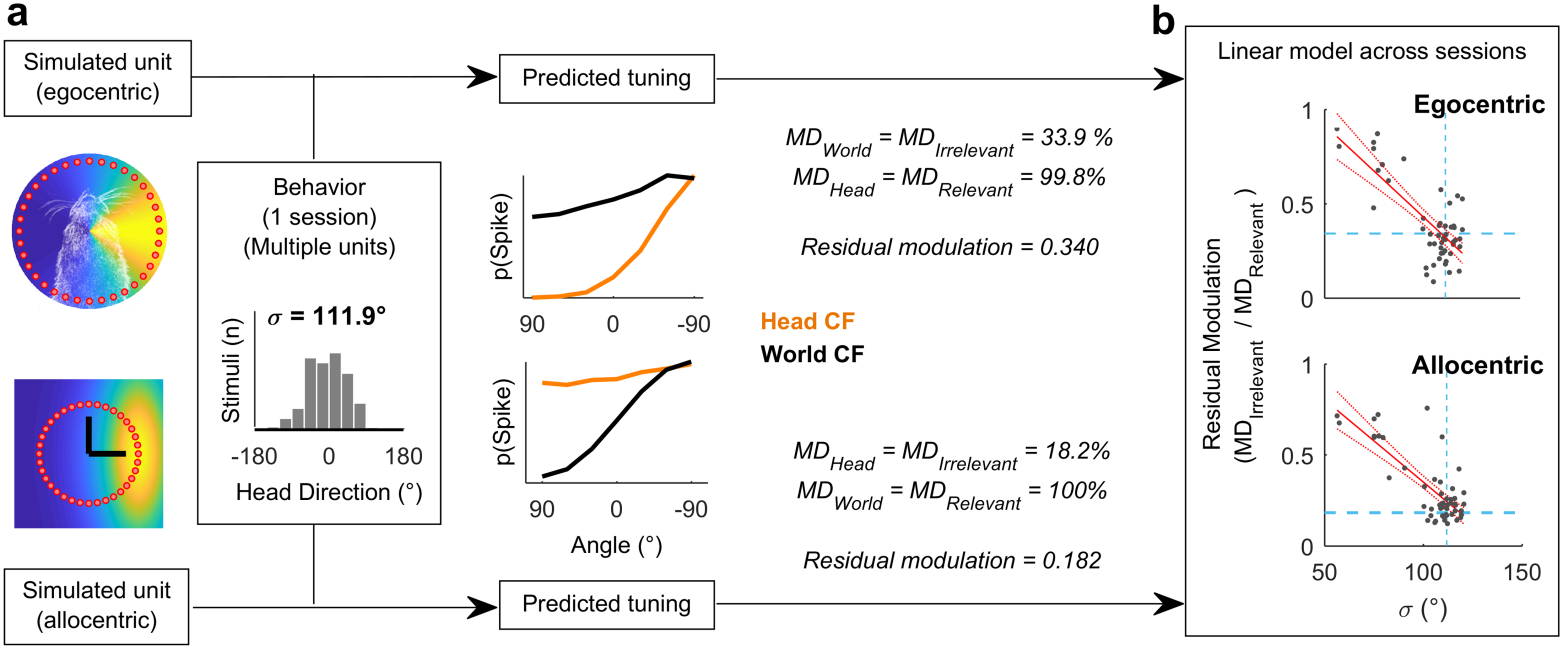
Estimating residual modulation. **a**: Example workflow for estimating residual modulation in coordinate frames irrelevant for neural output that result from biases in head direction. Residual modulation was defined as: (MD_Irrelevant_ / MD_Relevant_). Estimations performed separately using simulated units for each behavioral session. **b**: Residual modulation was inversely correlated with the standard deviation of head directions (σ). Red, filled lines indicate regression fit and confidence intervals. Dashed lines indicate the data point for the single session in (a). Data available at https://doi.org/10.6084/m9.figshare.4955291.v1.

To assess the influence of real animal behavior on our ability to distinguish coordinate frames, we combined our simulated egocentric and allocentric receptive fields (Fig 1) with the animal’s head position and direction across each single behavioral testing session (Fig 3a). This allowed us to calculate the spatial tuning for known allocentric and egocentric receptive fields in both head and world coordinate frames. Our simulation predictions (Fig 1) demonstrated that for a uniform distribution of head angles, the tuning function of allocentric or egocentric units should be flat when considered in the irrelevant coordinate frame. However, a bias in head location over time would produce spatial modulation in firing rate with location in the irrelevant coordinate frame (Fig 3a). In order to account for this, we therefore measured residual modulation as the ratio of modulation depth in each coordinate frame (Fig 3; MD_Irrelevant_ / MD_Relevant_). Residual modulation thus represents the degree of indirect spatial tuning in one coordinate frame observed as a by-product of the animal’s behavior combined with spatial tuning in another coordinate frame.

Across all behavioral sessions, residual modulation was inversely correlated with variation in the animal’s head direction (expressed as standard deviation) for both egocentric (R^2^ = 0.562, *p* = 1.07 x 10^−10^) and allocentric simulated units (R^2^ = 0.615, *p* = 3.73 x 10^−12^) (Fig 3b). This indicated that for real animal behavior, we would not expect to see the complete abolition of tuning but rather spatial tuning in both coordinate frames, with the weaker tuning potentially attributable to the animal’s bias in head direction. In our neural analysis, we thus used the relationship between behavior and residual modulation to provide a statistical framework in which to assess the significance of spatial tuning of real neurons.

### Egocentric and allocentric tuning in auditory cortex

During exploration, we recorded the activity of 186 sound-responsive units (50 single units, 136 multi-units) in auditory cortex (S2 Fig). Electrode arrays were targeted to span the low-frequency areas in which the middle and posterior ectosylvian gyral regions meet, and thus units were sampled from primary auditory cortex and two tonotopically organized secondary fields: the posterior pseudosylvian and posterior suprasylvian fields. We analyzed the firing rates of units in the 50 milliseconds after the onset of each click; this window was wide enough to capture the neural response while being sufficiently short so that the animal’s head moved less than 1 cm (median 4 mm, S3 Fig) and less than 30° (median 12.6°)—the interval between speakers. The time interval between stimuli always exceeded 250 ms.

**Fig 4:**
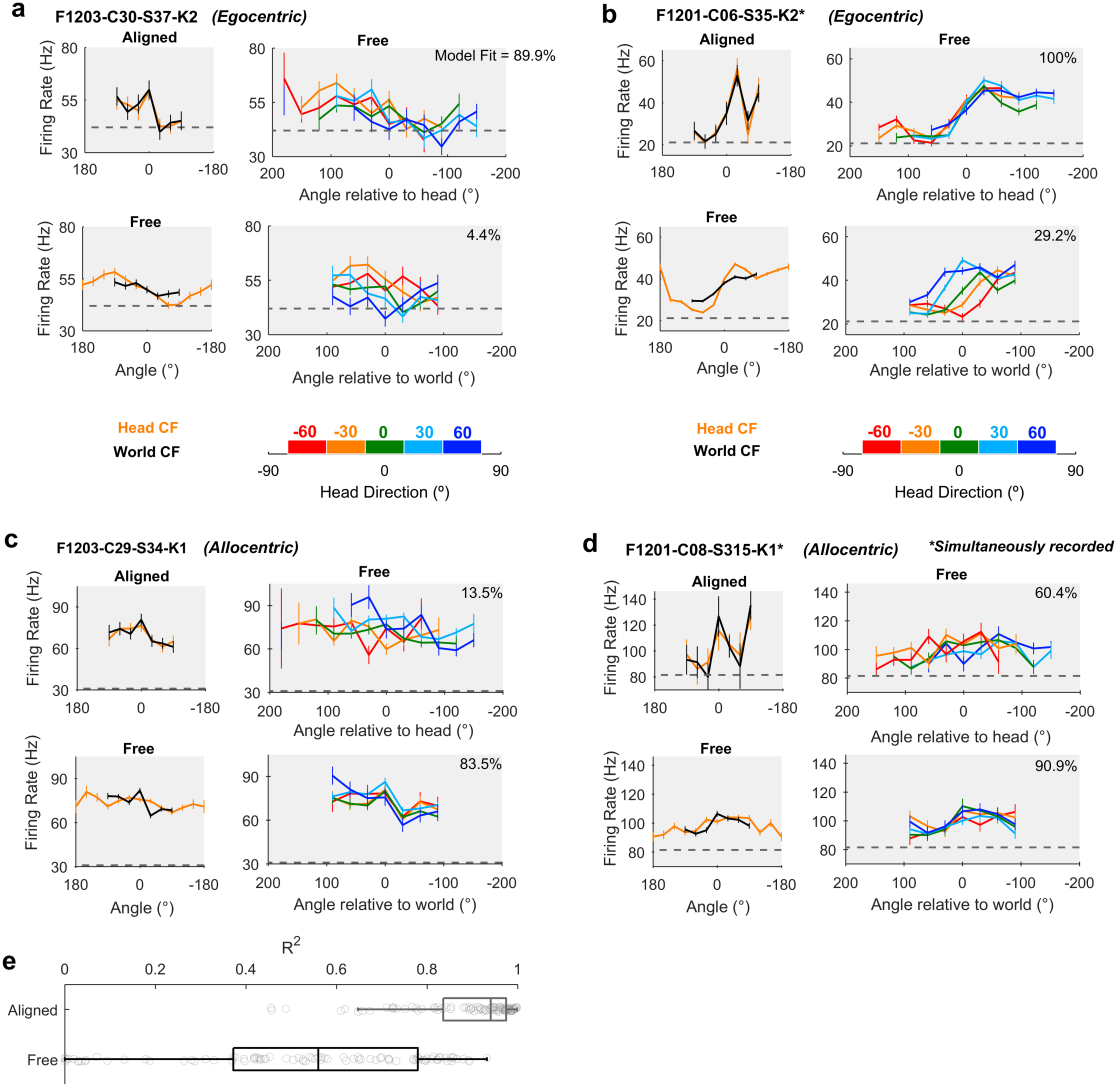
Spatial tuning of egocentric and allocentric units. **a-d**: Spatial tuning of four example units that were classified as egocentric (a-b) or allocentric (c-d). In each panel, top left: tuning curves calculated for sound angle in head and world coordinate frames (CFs) when both frames were aligned. Bottom left: tuning curves when head and world CFs were free to vary. Top and bottom right: tuning curves plotted at specific head rotations. Model fit refers to the percentage of explainable deviance calculated according to Fig 6 across all data in which coordinate frames were free to vary. Data for all tuning curves are shown as mean ± SEM firing rates. Dotted lines show the mean background activity measured in the 50 ms before stimulus presentation. **e**: Correlation coefficients between tuning curves in head and world CFs when aligned or free to vary as the animal foraged around the arena. Boxplots show median and inter-quartile range; symbols show coefficients for individual units. Data available at https://doi.org/10.6084/m9.figshare.4955300.v1.

We identified periods of time during which the animal was facing forwards (± 15° of the arena midline) at the center of the speaker ring (S4 Fig): in this situation, we mimic classic neurophysiological investigations of spatial tuning in which head and world coordinate frames are aligned. In the aligned case, we recorded 92 units that were significantly modulated by sound source location (Fig 4a–4d: Top left, general linear model [GLM] analysis of deviance, *p* ≤ 0.05) and for which spatial tuning curves computed in head and world coordinate frames were highly correlated (mean ± SEM: R^2^ = 0.889 ± 0.0131). We then compared the aligned control condition with all data in which the head and world coordinate frames were free to vary. Compared to the aligned condition, the correlation between egocentric and allocentric tuning curves when coordinate frames were free to vary was significantly lower (Fig 4e, R^2^ = 0.522 ± 0.0294) (paired *t* test: free versus aligned *t*_91_ = 8.76, *p* < 0.001), and differences in spatial tuning in head and world coordinate frames became visible (Fig 4a–4d: Bottom left).

When animals moved freely through the arena, and head and world coordinate frames were thus dissociated, we observed units consistent with egocentric (Fig 4a and 4b, S5 and S6 Figs) and allocentric hypotheses (Fig 4c and 4d, S7 and S8 Figs). For units consistent with the egocentric hypothesis, spatial receptive fields were more strongly modulated by sound angle in the head coordinate frame than the world coordinate frame. For the unit shown in Fig 4a, modulation depth values in the head and world coordinate frames were 28.3% and 10.1%, respectively. In Fig 4b, modulation depth was 49.0% in the head coordinate frame and 30.3% in the world coordinate frame. Furthermore, tuning curves for sounds plotted relative to the head remained consistent across head rotation but shifted systematically when plotted relative to the world (Fig 4a and 4b: Right columns). Both outcomes are highly consistent with our simulation predictions (Fig 1).

In addition to identifying head-centered spatial tuning across movement, we also found units with spatial tuning that realized the predictions generated by the allocentric hypothesis. These units showed greater modulation depth to sound angle in the world coordinate frame than the head coordinate frame (Fig 4c and 4d, S7 and S8 Figs): For putative allocentric units, modulation depths for tuning curves were 21.2% and 13.4% in the world and head coordinate frames, respectively, for the unit shown in Fig 4c and 12.7% and 10.1%, respectively, for the unit shown in Fig 4d. For allocentric units, spatial tuning in the world coordinate frame was robust to head rotation, whereas tuning curves expressed relative to the head were systematically shifted when mapped according to head direction (Fig 4c and 4d: Right column).

### Modulation depth across coordinate frames

**Fig 5:**
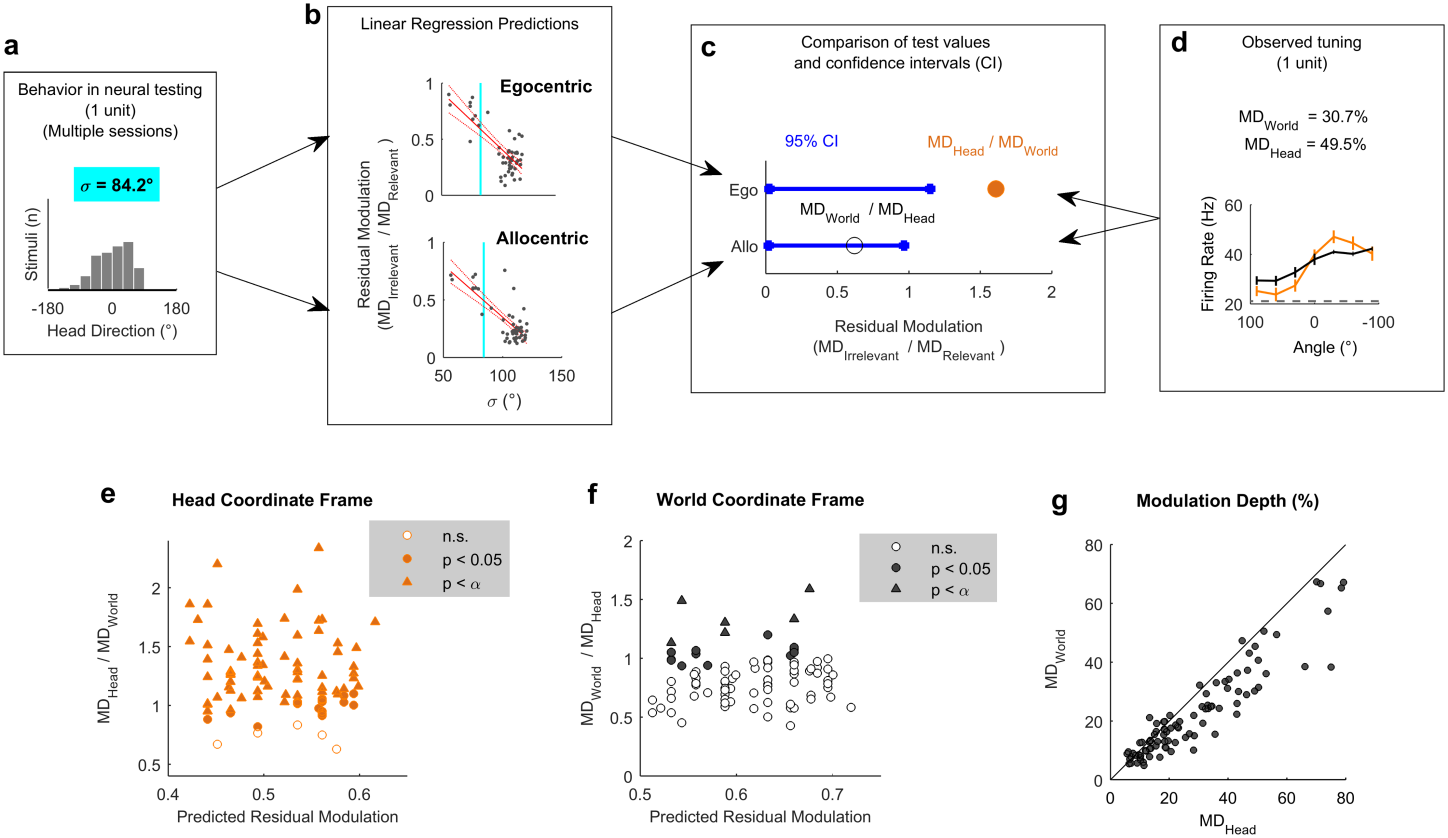
Modulation depth across coordinate frames. **a-d**: Workflow illustrating the use of animal behavior (a: summarized using the standard deviation of head directions during neural testing, σ) and linear regression models (b: see also Fig 3) to generate confidence intervals (CIs) for residual modulation (c) that were compared to observed modulation depth values (d), normalized relative to the alternative coordinate frame. Blue vertical lines in (b) show the σ value in (a). **e-f**: Normalized modulation depth observed for each spatially tuned unit compared against the mean residual modulation predicted from behavior in head (e) or world (f) coordinate frames. Bonferroni-corrected statistical criterion (*p* = 5.43 x 10^−4^) is denoted by α. **g**: Modulation depth for all spatially modulated units (*n* = 92) compared in world (MD_World_) and head coordinate frames (MD_Head_) during exploration. Data available at https://doi.org/10.6084/m9.figshare.4955342.v1.

To quantify the observations we made above and systematically compare spatial tuning in world and head coordinate frames, we calculated modulation depth for both tuning curves for each unit. We next asked if modulation depth observed in either head or world coordinate frames was greater than the residual modulation predicted by our earlier simulations (Fig 3b). A linear regression model developed using simulated receptive fields was used to predict the magnitude of residual tuning for each coordinate frame from the animal’s behavior during the recording of each unit (Fig 5a–5d). To describe the animal’s behavior across the relevant testing sessions for each neural recording, we calculated the standard deviation of head directions (Fig 5a). A smaller standard deviation indicates a less uniform range of head-directions and when combined with our regression model (Fig 5b) would predict higher residual modulation in both coordinate frames. Thus, for a given standard deviation, we could use linear regression to obtain a predicted confidence interval for the residual modulation in head and world coordinate frames arising from allocentric or egocentric tuning, respectively (Fig 5c). The observed modulation values were calculated for each unit as the ratio of modulation depth in one coordinate frame divided by the other coordinate frame (Fig 5d), and significance was attributed when test values exceeded the confidence interval of residual modulation.

Across all spatially tuned units, modulation in the head coordinate frame was significantly greater than predicted for 87/92 units (94.6%) (*p* < 0.05, Fig 5e); modulation in the world coordinate frame was significant for 19/92 units (20.7%, Fig 5f). For 14/92 units (15.2%), modulation depth was significantly greater than expected in both coordinate frames. When Bonferroni corrected for multiple (*n* = 92, α = 5.43 x 10^−4^) comparisons, these numbers dropped to 69/92 units (75%) for modulation in the head coordinate frame, none of which were additionally modulated in a world coordinate frame, and 6/92 units (6.5%) for modulation in the world coordinate frame—none of which showed significant head-centered modulation. With this more-conservative statistical threshold, modulation depths were not significantly greater than expected in either coordinate frame for the remaining 17/92 units (18.5%). Together these observations suggest that response types occupy a continuum from purely egocentric to purely allocentric. The existence of units with significant modulation in both coordinate frames with a less-conservative statistical cutoff, or no significant modulation with corrected threshold, may indicate mixed spatial sensitivity comparable with other reports in auditory cortex [23].

A key prediction from our simulations with both uniform head-directions (Fig 1) and actual head-directions (Fig 3a) was that modulation depth would be greater in the coordinate frame that was relevant for neural activity than the irrelevant coordinate frame (i.e., Head > World for egocentric; World > Head for allocentric). Having demonstrated that modulation within both co-ordinate frames was greater than expected based on the non-uniform sampling of head direction, we compared the modulation depth across coordinate frames for all spatially tuned units (Fig 5g). For 76/92 units (82.6%), we observed greater modulation depth in the head coordinate frame than the world coordinate frame, indicating a predominance of egocentric tuning and a minority of units (16/92, 17.4%) in which allocentric tuning was strongest.

### General linear modelling to define egocentric and allocentric populations

Our analysis of modulation depth indicated the presence of both egocentric and allocentric representations in auditory cortex but also highlighted that the analysis of modulation depth alone was sometimes unable to resolve the coordinate frame in which units encoded sound location. To calculate modulation depth requires that we discretize sound location into distinct angular bins, average neural responses across trials, and thus ignore single trial variation in firing rates. GLMs potentially offer a more-sensitive method, as they permit the analysis of single trial data and allow us to treat sound angle as a continuous variable. We considered two models, which either characterized neural activity as a function of sound source angle relative to the head (GLM_HEAD_) or in the world (GLM_WORLD_). For all units for which at least one GLM provided a statistically significant fit (relative to a constant model, analysis of deviance; *p* < 0.05, 91/92 units), we compared model performance using the Akaike information criterion (AIC)[24] for model selection. In accordance with the modulation depth analysis, the majority of units were better modelled by sound angle relative to the head than world (72/91 units; 79.1%; 4 animals), consistent with egocentric tuning. However, we also observed a smaller number of units (19/91 units; 20.9%; 3 animals) whose responses were better modelled by sound angle in the world and thus showed stronger representation of allocentric sound location.

**Fig 6:**
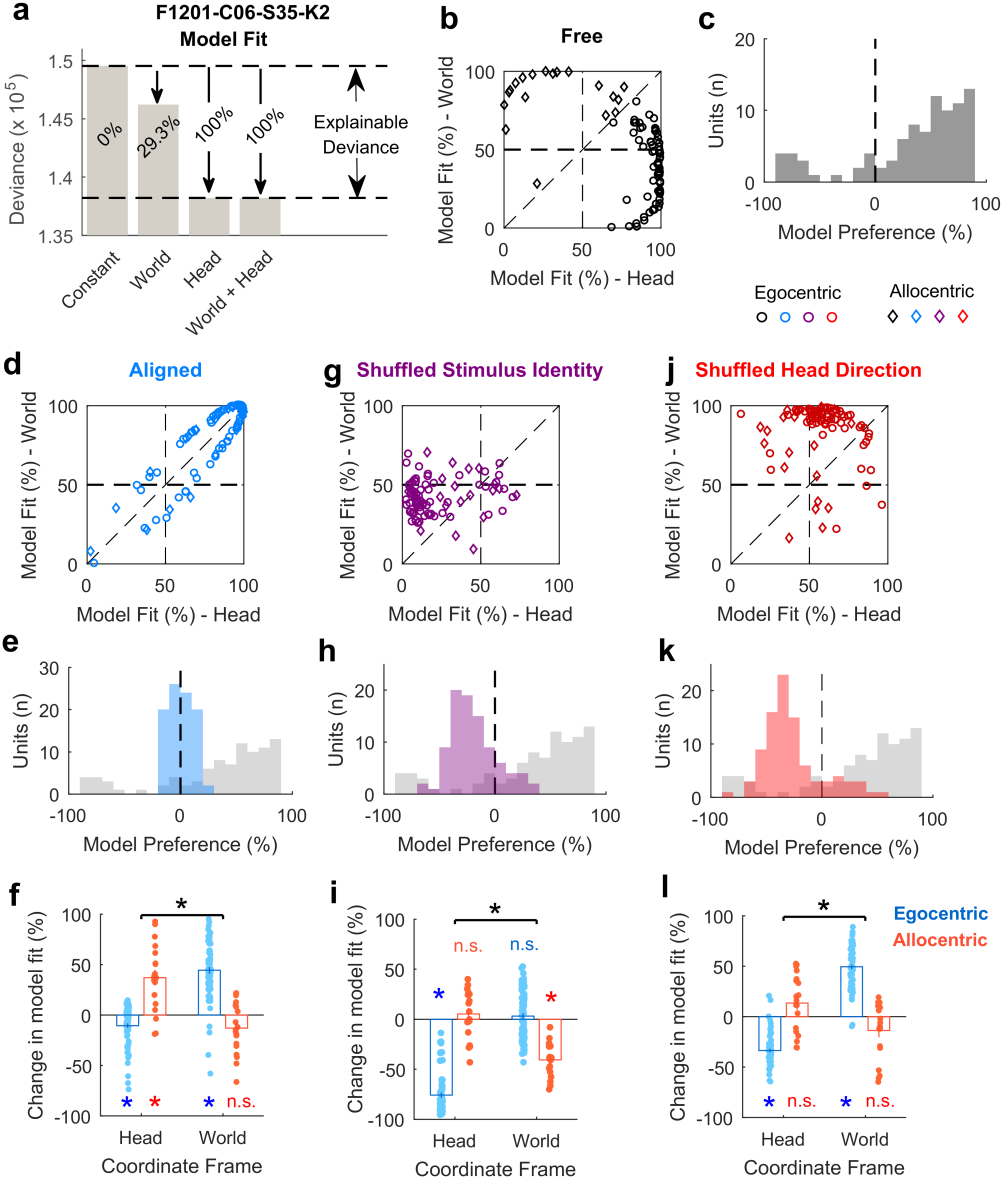
General linear modelling of spatial sensitivity. **a**: Calculation of model fit for sound angle relative to the head or world. Raw deviance values were normalized as a proportion of explainable deviance; the change in deviance between a constant and a full model. **b**: Model fit comparisons for all units when the animal was free to move through the arena. **c**: Model preference that indicates the distribution of units across the diagonal line of equality in (b). **d-e**: Model fit and model preference for data when the head and world coordinate frames were aligned. The grey background in (e) shows the distribution of model preference in the freely varying condition for reference. **f**: Change in model fit between freely moving and aligned states for egocentric and allocentric units in head and world coordinate frames. **g-h**: Model fit and model preference for freely moving data when speaker identity was shuffled. Data points in (g) show for each unit the median model fit averaged across 1,000 shuffles. **i**: The change in model fit between unshuffled and shuffled data. **j-k**: Model fit and model preference for freely moving data when the animal’s head direction was shuffled. Data points in (j) show for each unit median model fit averaged across 1,000 shuffles. **l**: The change in model fit between unshuffled and shuffled data. Asterisks with horizontal bars in (f), (i), and (l) indicate significant interactions (two-way anova on change in model fit with shuffle) between coordinate frame (head/world) and unit type (egocentric/allocentric) (*p* < 0.001). Asterisks/n.s. for each bar represent significant/non-significant effects of shuffle on model fit in specific coordinate frames and for specific unit types (red/blue; *t* test, *p* < 0.05). Data available at https://doi.org/10.6084/m9.figshare.4955360.v1.

To visualize GLM performance and explore egocentric and allocentric tuning further, we plotted a normalized metric of the deviance value usually used to assess model fit. Here, we defined **model fit** as the proportion of explainable deviance (Fig 6a) in which a test model (e.g., GLM_WORLD_) is considered in the context of GLMs that have no variable predictors of neural activity (a constant model) or use sound angle in both coordinate frames as predictors (a full model). This normalization step is critical in comparing model fit across units, as deviance values alone are unbounded. In contrast, model fit is limited from 0% (indicating the sound angle provides little information about the neuron’s response) to 100% (indicating the sound angle in one coordinate frame accounts for the neuron’s response as well as sound angles in both frames). While the units we recorded formed a continuum in this space, for the purpose of further analysis we defined egocentric and allocentric units according to the coordinate frame (head/world) that provided the best model fit as determined by the AIC above.

Using a GLM-based analysis, we predicted that egocentric units would have a high percentage of the full model fit by sound angles relative to the head and a low model fit for sound angles relative to the world and that allocentric units would show the reciprocal relationship. To test these predictions, we generated a **model preference** score; the model fit for sound angles relative to the head minus the model fit for sound angles in the world. Accordingly, negative values of model preference should identify allocentric units, while positive values should indicate egocentric units. Neurons in which both sound angles relative to the world and head provide high model fit values may represent sounds in intermediary or mixed coordinate frames and would have model preference scores close to zero, as would neurons in which we were unable to disambiguate coordinate frame preference due to non-uniform head angle distributions.

In the space defined by model fit for sound angles relative to the head and world (Fig 6b), units clustered in opposite areas, supporting the existence of both egocentric and allocentric representations. This clustering was also evident in the model preference scores, which showed a bimodal distribution (Fig 6c). Repeating this analysis on data in which the head and world coordinate frames were aligned (due to the animals’ position at the center of the speaker ring) demonstrated that model fit values for head and world coordinate frames became more similar, and model preference scores were centered around zero (Fig 6d and 6e). When we compared the change in model fit with alignment (two-way anova), this was reflected as a significant interaction between coordinate frame (head or world) and unit type (egocentric or allocentric, determined by the coordinate frame that provided best model fit using the AIC, *F*_1,178_ = 130.1, *p* = 5.71 x 10^−23^). Post hoc comparison confirmed that model fit in the head coordinate frame decreased significantly for egocentric units (Bonferroni corrected for multiple comparisons, *p* = 5.04 x 10^−5^) and increased significantly for allocentric units (*p* = 4.36 x 10^−4^). In contrast, in the world coordinate frame, alignment led to a significant increase in model fit for egocentric units (*p* = 2.22 x 10^−20^) and a non-significant decrease in model fit for allocentric units (*p* = 0.155).

We performed two additional control analyses on the freely moving dataset: firstly, we randomly shuffled the speaker identity while maintaining the same information about the animal’s head direction. Randomizing the speaker identity should affect the ability to model both egocentric and allocentric neurons, and we would therefore predict that model fits for spatially relevant coordinate frames would be worse, and model preference scores would tend to zero (i.e., shuffling would shift model preference scores in the negative direction for egocentric units and the positive direction for allocentric units). As predicted, shuffling speaker identity eliminated clustering of egocentric and allocentric units in the space defined by model fit (Fig 6g) and led to opposing effects on model preference (Fig 6h and 6i): Model fit scores for egocentric and allocentric units were both affected by shuffling speaker identity but in opposite directions (unit x coordinate frame interaction, *F*_1, 178_ = 227.4, *p* = 1.22 x 10^−33^). For egocentric units, the model fit for sound angle relative to the head declined significantly with shuffling (*p* = 7.44 x 10^−41^), while fit for sound angle in the world did not change significantly (*p* = 0.271). For allocentric units, model fit for sound angle in the world declined significantly (*p* = 1.29 x 10^−8^) but was not significantly different in the head coordinate frame (*p* = 0.35). When shuffling stimulus angle (averaging across 1,000 shuffles), we identified 12/19 (63.2%) allocentric and 63/72 (87.5%) egocentric units with model preference scores beyond the 97.5% limits of the shuffled distribution.

Secondly, we shuffled information about the animal’s head direction while maintaining information about speaker identity. This should cause model fit values to decline for sound angle relative to the head for egocentric units and should therefore result in egocentric units shifting their model preference scores towards zero. For allocentric units, the model fit for sound location in the world should be maintained, and we would not predict a systematic change in model preference. This was indeed the case (Fig 6j–6l; interaction between coordinate frame and unit type: *F*_1, 178_ = 216.7, *p* = 1.35 x 10^−32^): For egocentric units shuffling head direction significantly reduced model fit in the head coordinate frame (*p* = 1.40 x 10^−27^) and increased model fit in the world coordinate frame (*p* = 2.20 x 10^−31^). For allocentric units, shuffling head direction did not significantly affect model fit in either head (*p* = 0.211) or world coordinate frames (*p* = 0.178).

### Egocentric and allocentric units—Population characteristics

**Fig 7:**
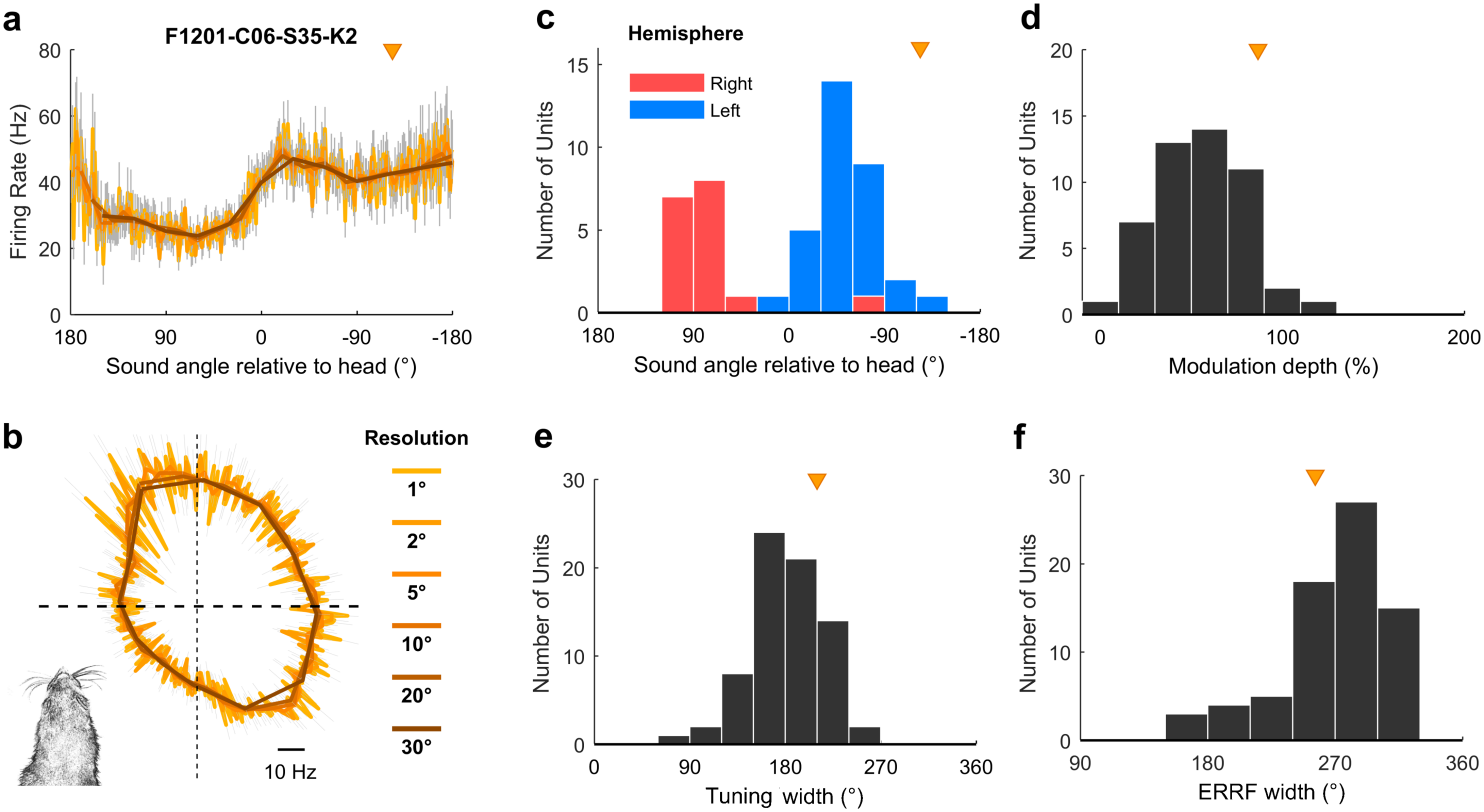
Egocentric unit characteristics. **a-b**: Spatial tuning of an example egocentric unit at multiple angular resolutions. Data are shown as mean ± SEM firing rate plotted in Cartesian (a) or polar (b) coordinates. Triangle indicates the preferred location of unit. Inset (b) illustrates the corresponding head direction onto which spatial tuning can be super-imposed. **c**: Preferred location of all egocentric units (**n** = 72) in left and right auditory cortex. **d**: Modulation depth calculated according to [18] across 360° for units in both hemispheres. **e-f**: Tuning width (e) and equivalent rectangular receptive field (ERRF) width (f) for all units. Triangle indicates the preferred location, modulation depth, tuning width, and ERRF of the example unit in (a). Data available at https://doi.org/10.6084/m9.figshare.4955366.v1.

For egocentric units that encoded sound location in the head coordinate frame, it was possible to characterize the full extent of tuning curves in 360° around the head (Fig 5a and 5b), despite our speaker array only spanning 180°. This was possible because the animal’s head direction varied continuously across 360° and so removed the constraints on measurement of spatial tuning imposed by the range of speaker angles used. Indeed, we were able to extend our approach further to characterize super-resolution tuning functions with an angular resolution more precise than the interval between speakers (Fig 7a and 7b and S9 Fig). Together these findings show it is possible to use changes in head direction to recover the spatial tuning of individual units with greater detail than would be possible if the subject’s head position and direction remained constant relative to the sound sources. Egocentric units shared spatial receptive field properties typical of previous studies [15,16,18,25]: units predominantly responded most strongly to contralateral space (Fig 7c) with broad tuning widths (Fig 7e and 7f) that typify auditory cortical neurons. We also found similar, if slightly weaker, spatial modulation when calculating modulation depth according to Ref. [18] (Fig 7d).

**Fig 8:**
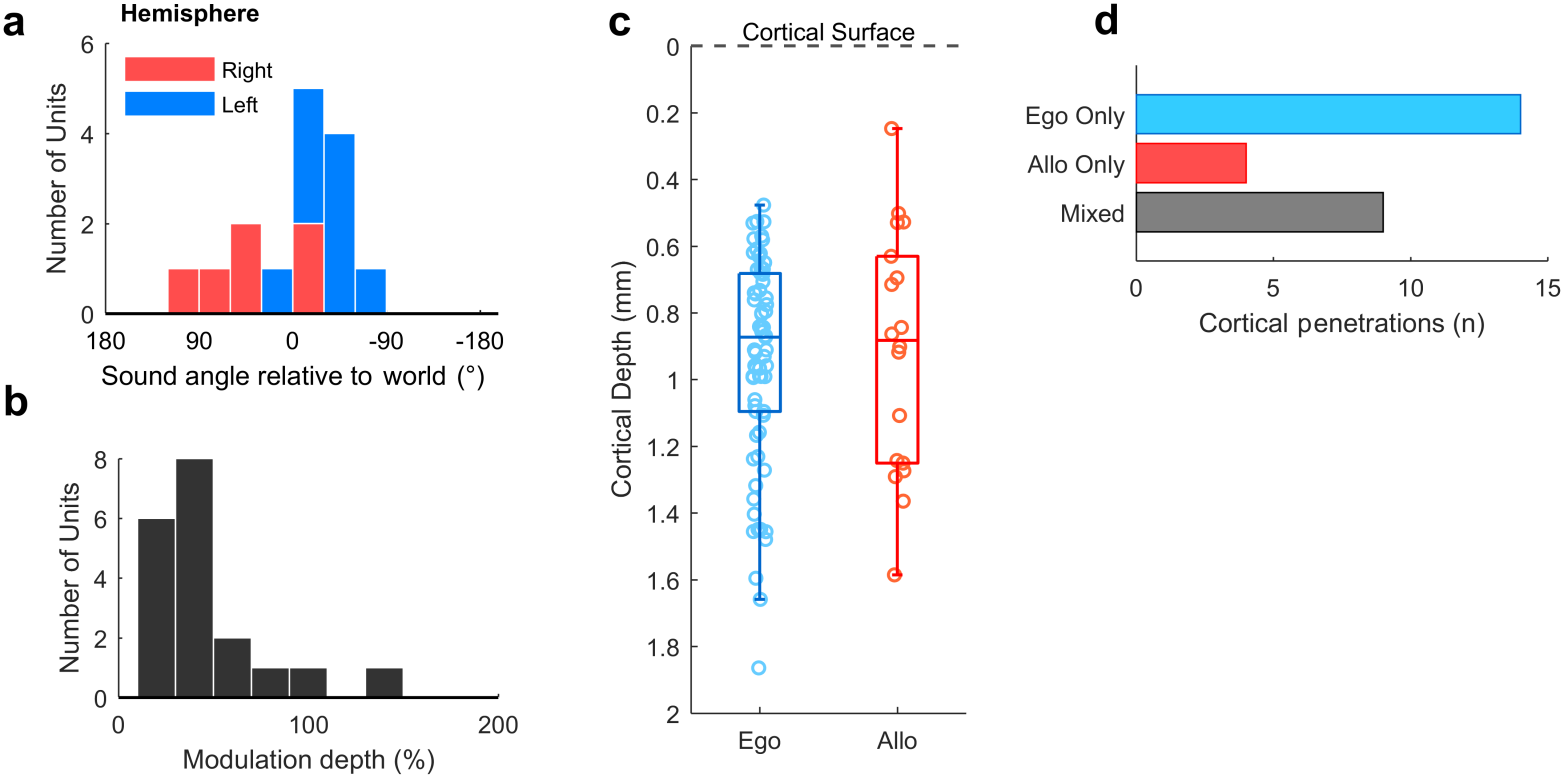
Allocentric unit characteristics. **a**: Preferred location of all allocentric units (*n* = 19) in left and right auditory cortex. **b**: Modulation depth calculated across 180° for units in both hemispheres. **c**: Comparison of cortical depth at which egocentric and allocentric units were recorded. Ferret auditory cortex varies in thickness between 1.5 mm and 2 mm, and electrode depths were confirmed histologically (S2 Fig). **d**: Number of cortical penetrations on which we recorded only egocentric units, only allocentric units, or a combination of both (mixed). All 92 spatially tuned units were recorded on 27 unique electrodes, with recorded units being distributed throughout cortex as the electrodes were descended. Data available at https://doi.org/10.6084/m9.figshare.4955369.v1.

For allocentric units, we observed a similar contralateral tuning bias in preferred location (Fig 8a) to egocentric units and that allocentric units had relatively low modulation depths (Fig 8b). These tuning features may not be surprising, as an allocentric receptive field could presumably fall anywhere within or beyond the arena and might therefore be poorly sampled by circular speaker arrangements. If the tuning curves measured here were in fact sampling a more complex receptive field that related to a world-centered coordinate frame (Fig 1b), then we would predict that the receptive fields would be correspondingly noisier. Allocentric and egocentric units were recorded at similar cortical depths (Fig 8c) and on the same cortical penetrations; egocentric units were recorded on 9 of 13 electrodes (69.2%) on which we also identified allocentric units (Fig 8d).

### Timing of spatial information

**Fig 9:**
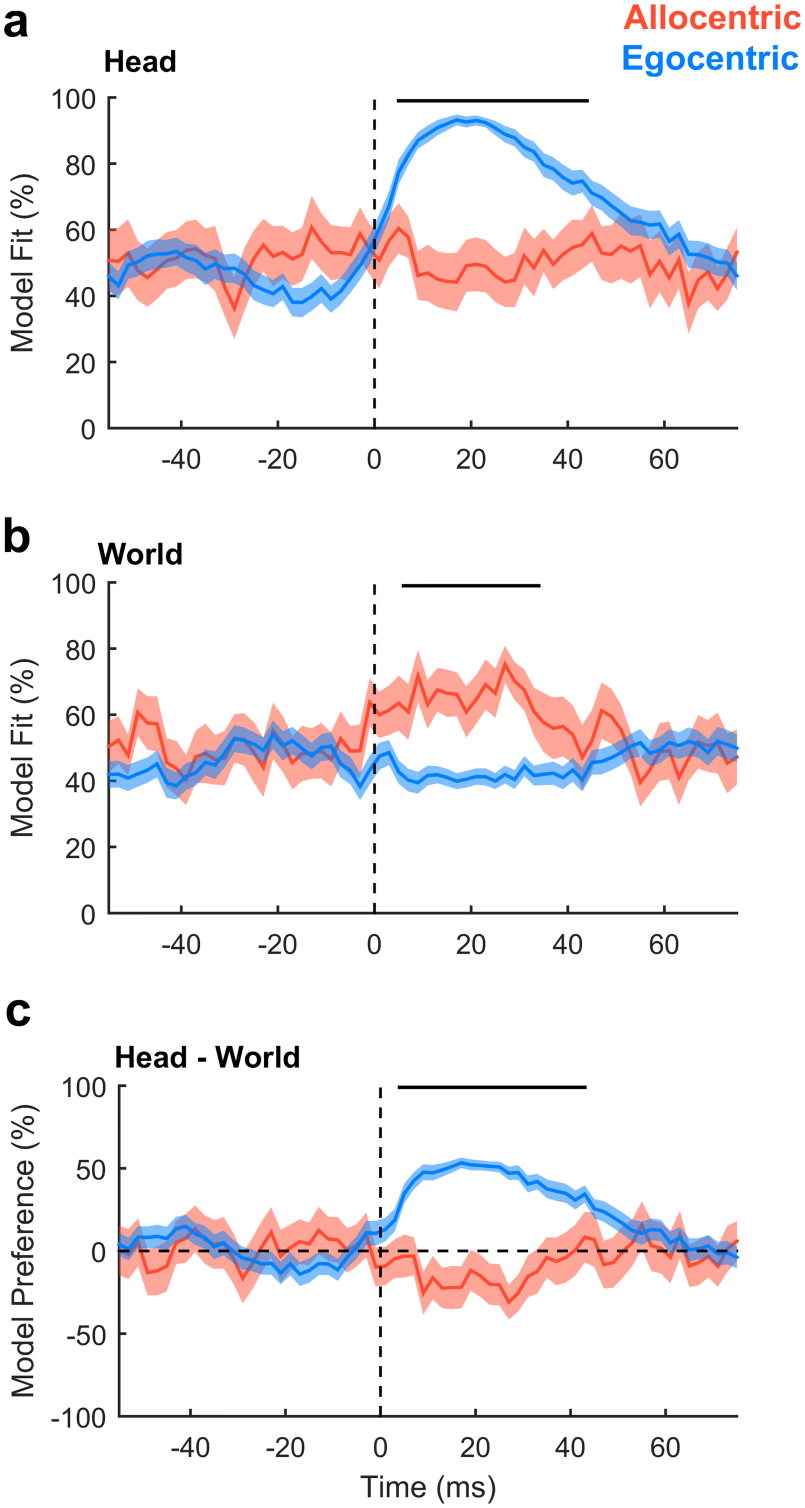
Egocentric and allocentric tuning over time. **a**: Model fit for predicting neural activity from sound angles relative to the head. **b**: Model fit for predicting neural activity from sound angles in the world. **c**: Model preference. Data are shown as mean ± SEM for egocentric and allocentric unit populations. Black lines indicate periods of statistical significance (cluster-based unpaired *t* test, *p* < 0.05). Data available at https://doi.org/10.6084/m9.figshare.4955372.v1.

Our findings suggested the existence of egocentric and allocentric tuning in auditory cortex. As these descriptions were functionally defined, we hypothesized that differences between allocentric and egocentric tuning should only arise after stimulus presentation. To test this, we analyzed the time course of unit activity in a moving 20 ms window and compared model fit and model preference of egocentric and allocentric units (defined based on the AIC analysis above) using cluster-based statistics to assess significance [26]. Model fit for sound angles relative to the head was greater for egocentric than allocentric units only between 5 ms and 44 ms after stimulus onset (Fig 9a, *p* = 0.001996). Model fit for sound angles in the world was greater for allocentric than egocentric units only between 6 ms and 34 ms after stimulus (Fig 9b, *p* = 0.001996). Model preference diverged only in the window between 4 ms and 43 ms after stimulus onset (Fig 9c, *p* = 0.001996). We observed no differences between egocentric and allocentric units before stimulus onset or when coordinate frames were aligned (S10 Fig). Thus, the differences between egocentric and allocentric units reflected a stimulus-evoked effect that was only observed when head and world coordinate frames were free to vary.

### Population representations of space

**Fig 10:**
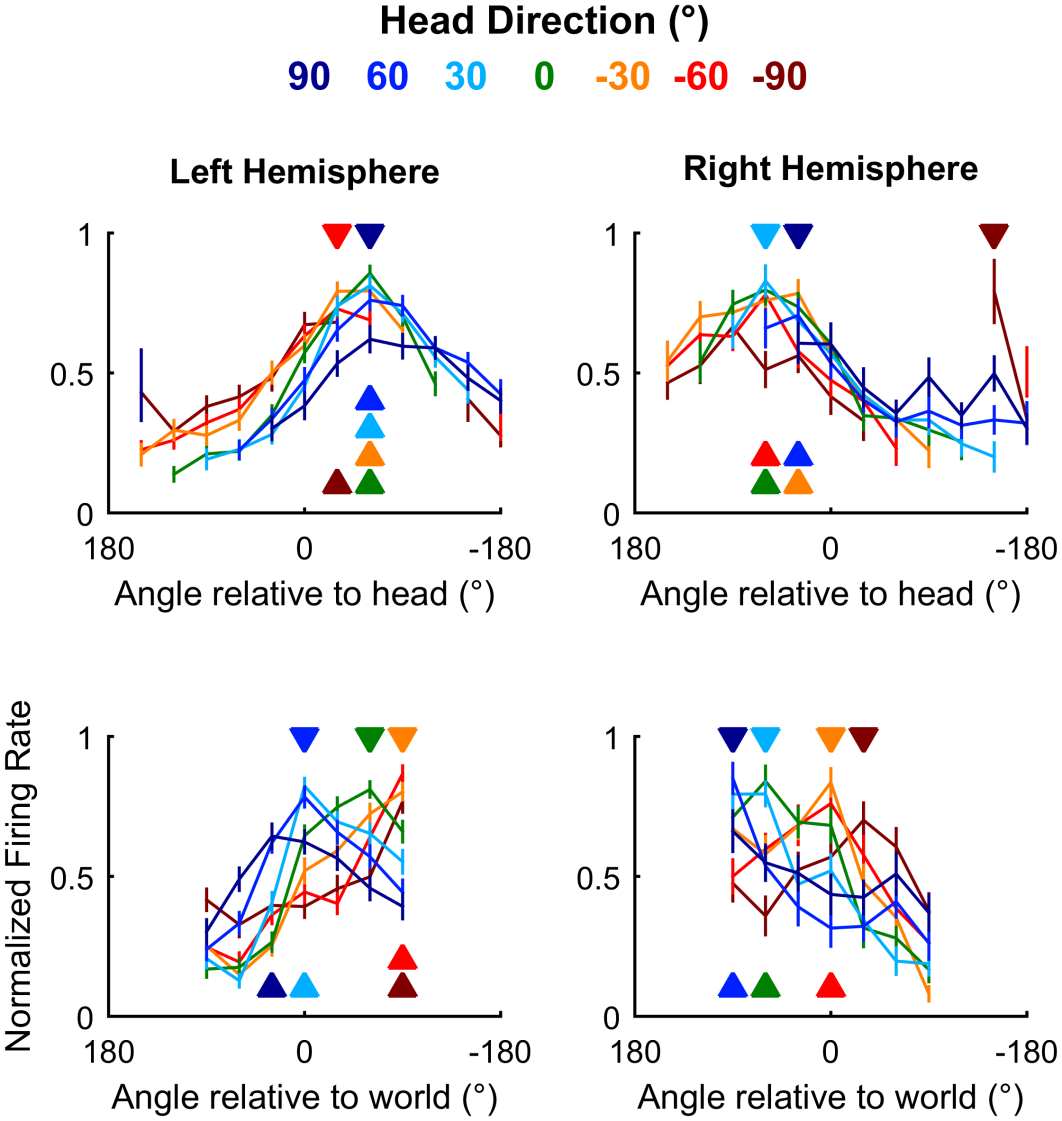
Auditory cortical tuning. Population tuning curves plotted across head direction for mean (±SEM) normalized response of all units in left and right auditory cortex; filled triangles indicate sound angle of maximum response at each head direction. Data available at https://doi.org/10.6084/m9.figshare.4955222.v1.

We next asked how auditory cortical neurons behaved as a population when spatial tuning was compared across head directions. In contrast to individual units, population activity more closely reflects the large-scale signals observed in human studies using EEG to distinguish coordinate frame representations [11,12]. To form populations, we took the unweighted mean of normalized firing rates from all units recorded from left (*n* = 64) or right (*n* = 28) hemispheres and compared tuning curves measured at different head directions. As would be expected from the predominance of egocentric units, we found that tuning curves for both left and right auditory cortical populations were consistent within the head coordinate frame but not the world coordinate frame (Fig 10). Thus, the allocentric units we find here were sufficiently rare as to be masked in overall population readouts of spatial tuning, potentially accounting for conflicting findings of coordinate frame representations from EEG recordings.

### Distance modulation of cortical neurons and spatial tuning

Studying auditory processing in moving subjects allowed us to resolve coordinate frame ambiguity so that we could determine the spaces in which neurons represent sound location. However, recording in freely moving subjects also made it possible to go beyond angular measurements of the source location and address how neurons represented the distance of sound sources. Though often overlooked, distance is a critical component of egocentric models of neural tuning, as the acoustic cues indicating sound location such as inter-aural level differences (ILDs) change as sound sources approach the head. For distant sound sources (typically > 1 m), ILDs are small relative to distance-related attenuation of sound; however, ILDs become much larger as sounds approach the head, and source-receiver distance decreases [27,28]. Neurons must therefore accommodate for distance-dependent cues to accurately represent sound location across changes in head position. In our study, the distance between head and sound source ranged from 10 cm (the minimum distance imposed by the arena walls) to 40 cm, with only 3.37% of stimuli (mean across 57 test sessions) presented at greater distances (Fig 2g), and thus stimuli were likely to produce large ILDs [27,28].

**Fig 11:**
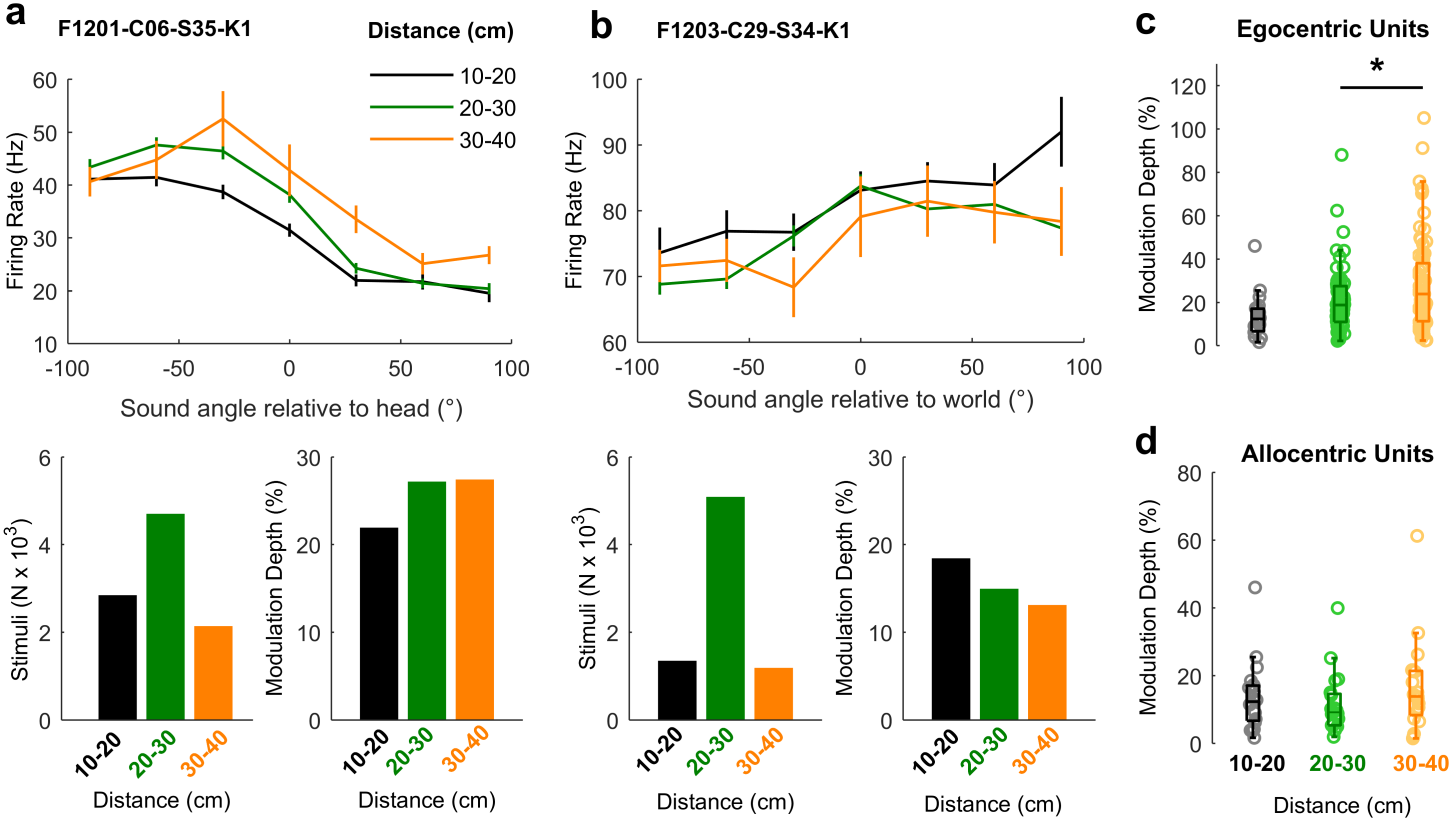
Spatial tuning across distance. **a-b**: Tuning curves of an egocentric (a) and allocentric (b) unit obtained with sound sources at varying distances from the animal’s head. Bar plots show the number of stimuli and modulation depth for each tuning curve. **c-d**: Distributions of modulation depth measured across distance for egocentric and allocentric units. Asterisk indicates significant pair-wise comparison (Tukey-Kramer corrected, **p** < 0.05). Data available at https://doi.org/10.6084/m9.figshare.4955375.v1.

Spatial tuning was observed at all distances studied in both egocentric (Fig 11a) and allocentric units (Fig 11b); however, modulation depth increased with distance for egocentric units (ANOVA, F_2,216_ = 3.45, *p* = 0.0334). Pairwise post hoc comparisons showed that modulation depth was largest for sounds at the greatest distances (Fig 11c), though the only significant difference was found for sounds presented 20 cm to 30 cm and 30 cm to 40 cm away (*t*_*72*_ = −3.54, *p* = 0.0279). In contrast, modulation depth did not change significantly with distance for allocentric units (Fig 11d, F_2, 57_ = 0.962, *p* > 0.1).

### Speed modulation of cortical neurons and spatial tuning

Changes in head position and direction also allowed us to investigate how speed of head movement (Fig 2f) affected neural activity. Movement is known to affect auditory processing in rodents [29–31], but its effects on spatial representations of sound location and also on auditory cortical processing in other phyla such as carnivores remain unknown. Here, we presented click sounds for which dynamic acoustic cues would be negligible, and thus, we could isolate the effects of head movement on neural activity.

**Fig 12:**
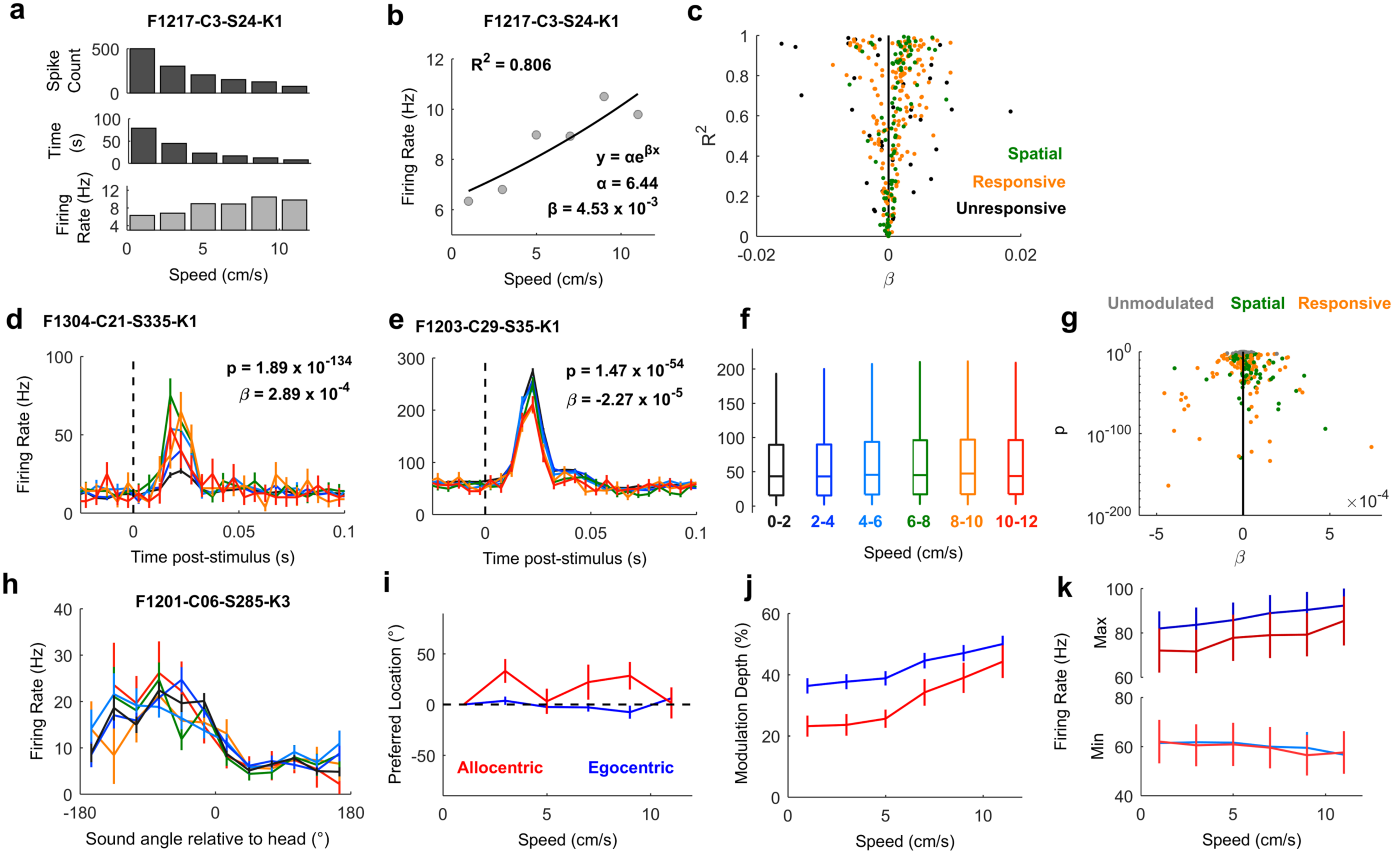
Speed-related auditory cortical activity and sensory processing. **a**: An example calculation of speed-related modulation of baseline firing of 1 unit using reverse correlation. **b**: An example speed-firing rate function summarized using regression (β) and correlation (R^2^) coefficients for the same unit as (a). **c**: Population distribution of regression and correlation coefficients showing the predominance of units with increasing speed-rate functions (β > 0). **d-e**: Peri-stimulus time histogram of sound-evoked responses for units that were modulated by speed: In (d), activity was enhanced as speed increased from 1 cm s^−1^ to 7 cm s^−1^ and decreased thereafter. In (e), firing decreased with increasing speeds, although speed-related modulation was smaller relative to sound-evoked activity than (d). **f**: Box plots showing distributions (median and inter-quartile range) of evoked firing rates in response to clicks across speed for all sound-responsive units. **g**: Population distribution of regression coefficients (β) and model fit (analysis of deviance *p* values) for all sound-responsive units. Units for which speed was not a significant predictor of neural activity (*p* > 0.001) are denoted in grey. **h**: Spatial tuning curve for one unit for clicks presented at different head-movement speeds. **i-k**: Change in preferred location (i), modulation depth (j), and min/max firing rates (k) of egocentric and allocentric units as a function of speed. Data for d-e and h-k are shown as mean ± SEM. Data available at https://doi.org/10.6084/m9.figshare.4955378.v1.

To address auditory cortical processing, first, we asked how many of our recorded units (regardless of auditory responsiveness or spatial modulation) showed baseline activity that varied with speed. For each unit, we took all periods of exploration (excluding the 50 ms after each click onset) and calculated the speed of the animal at the time of each action potential. We then discretized the distribution of spike-triggered speeds to obtain spike counts as a function of speed and normalized spike counts by the duration over which each speed range was measured. This process yielded a speed-rate function for baseline activity (Fig 12a). We then fitted an exponential regression curve to each function (Fig 12b) and plotted the correlation (R^2^) and regression coefficients (β) of each curve to map the magnitude and direction of association between speed and baseline activity (Fig 12c). Across the recorded population, we saw both positive and negative correlations representing units for which firing rate increased or decreased, respectively, with speed. However, a significantly larger proportion of the population increased firing rate with speed across all units (*t* test versus 0; *t*_308_ = 3.77, *p* = 1.97 x 10^−4^). This was also true if we considered only sound-responsive units (*t*_267_ = 5.15, *p* = 5.17 x 10^−7^) or only spatially tuned units (*t*_91_ = 4.12, *p* = 8.41 x 10^−5^).

We also observed speed-related modulation of sound-evoked responses in individual units (Fig 12d and 12e). For each individual unit, we characterized the relationship between head speed and single trial firing rates (averaged over the 50 ms post-stimulus onset) using a GLM that measured both the strength (analysis of deviance, *p* value) and direction (model coefficient, β) of association. Positive β values indicated an increase in firing rate with increasing speed, whereas negative β values indicated a fall in firing rate with increasing speed. Thus, the direction of the relationship between firing rate and speed was summarized by the model coefficient, allowing us to map the effects of movement speed across the population (Fig 12f). For 199/268 sound-responsive units (74.3%), speed was a significant predictor of firing rate (analysis of deviance versus a constant model, *p* < 0.001); however, the mean coefficient value for movement-sensitive units did not differ significantly from zero (*t*_199_ = 0.643, *p* = 0.521). This suggests that the sound-responsive population was evenly split between units that increased or decreased firing with speed. We noted that a significantly greater proportion of spatially modulated units (74/92, 80.4%) had sound-evoked responses that were sensitive to speed than units that were either not spatially modulated or for which we had insufficient sample sizes to test spatial modulation (125/176, 71.0%, Chi-squared test, *χ*^*2*^ = 3.96, *p* < 0.05). For those 74 spatially modulated and speed-sensitive units, coefficients were mostly larger than zero (mean ± SEM = 2.73 x 10^−5^ ± 1.53 x 10^−5^); however, this effect was not statistically significant (*t*_73_ = 1.80, *p* = 0.076). For the remaining speed-sensitive units, the mean coefficients was closer to zero (mean ± SEM = −5.02 x 10^−6^ ± 1.49 x 10^−5^).

Lastly, we asked if speed affected spatial tuning. Spatial tuning could be observed at all speeds of movement in spatially tuned units (Fig 12h), and the preferred locations of units did not vary systematically with speed (Fig 12i). For neither egocentric nor allocentric units was there a significant effect of speed in an ANOVA on preferred locations (egocentric: *F*_5, 432_ = 0.53, *p* = 0.753; allocentric: *F*_5, 108_ = 1.53, *p* = 0.188). However, we did observe significant changes in modulation depth (Fig 12j) both for egocentric (*F*_5, 432_ = 4.91, *p* = 0.0002) and allocentric units (*F*_5, 108_ = 5.09, *p* = 0.0003), indicating that spatial modulation was greater when the head was moving fastest. Change in modulation depth resulted from both a gradual suppression in minimum firing rates and enhancement in maximum firing rates with speed (Fig 12k). However, none of these changes in minimum or maximum firing rate were significant in comparisons across speed (ANOVA with speed as the main factor, *p* > 0.5), indicating that it was only through the aggregative change in responses to both preferred and non-preferred locations that modulation depth increased with speed.

## Discussion

Here we have shown that by measuring spatial tuning curves in freely moving animals, it is possible to demonstrate the coordinate frames in which neurons define sound location. For the majority of spatially sensitive auditory cortical neurons, we found egocentric tuning that confirms the broadly held but untested assumption that within the central auditory pathway, sound location is represented in head-centered coordinates. We also identified a small number of units with allocentric tuning, whose responses were spatially locked to sound location in the world, suggesting that multiple coordinate frames are represented at the auditory cortical level. These units were consistently identified across different analyses, observed in several subjects, and could be dissociated from simultaneously recorded egocentric receptive fields during the same behavioral sessions. Finally, we explored the dependence of neural activity and spatial tuning on sound source distance and the speed of head movement, demonstrating that both factors can modulate firing rates and spatial tuning in auditory cortex.

Our results show that an animal’s movement can be successfully tracked to measure head-centered egocentric tuning during behavior. Although we used speakers placed at 30° intervals across a range of 180°, we were nonetheless able to characterize spatial tuning of egocentric units around the full circumference of the head (i.e., Fig 7b). This illustrates the practical benefit of using moving subjects to characterize head-centered spatial tuning, as the animal’s head rotation generates the additional variation in sound angle relative to the head necessary to fully map azimuthal tuning with a reduced number of sound sources. Furthermore, as the animal’s head direction was continuous, the stimulus angle was also continuous, and thus, it was possible to measure spatial tuning at finer resolutions than that of the speaker ring from which stimuli were presented. In contrast to egocentric tuning, our ability to map allocentric receptive fields was limited by the speaker arrangement that only sparsely sampled world coordinates (Fig 2a). This may, in part, explain the low number of allocentric units in our population, and denser sampling of the world may reveal unseen allocentric tuning—for example, in the 50.5% (94/186) of units in which we recorded sound-evoked responses that were not spatially modulated. While a full 360° speaker ring may offer a minor improvement in sampling density, the radial organization of the ring remains a suboptimal design for sampling rectangular or irregular environments. To fully explore the shape of allocentric receptive fields will require denser, uniform speaker grids or lattices in environments through which animals can move between sound sources.

A notable property of egocentric units was the relationship between modulation depth of spatial tuning and distance, which was absent in allocentric units. This distance sensitivity may largely be driven by changes in ILDs, although other auditory and non-auditory cues can affect distance perception [32–36]. However, modulation depth in our study was lowest for proximal sounds when ILDs would be larger and when other cues such as inter-aural time differences remain relatively constant [27,28]. Localization of nearby sound sources (< 1 m) is possible for ferrets and humans [37,38], though within the range of distances we considered here, angular error of azimuthal localization in humans increases slightly as sounds approach the head [37]. Thus, our findings are consistent with human psychophysical performance but suggest that larger localization cues may not always produce better sound localization by neurons in auditory cortex. In the future, it will be critical to validate our findings for sound sources at greater distances where sound localization has been more widely studied.

In addition to recording many egocentric units, we also recorded a small number of allocentric units, supporting the idea that auditory cortex represents sound location in multiple coordinate frames [23] and parses an auditory scene into distinct objects [39,40]. We believe this is the first study to look for world-centered encoding of sound locations at the cellular level. Thus, the units we recorded, while small in number, reflect a novel spatial representation in the auditory system.

A key question is where allocentric units reside in cortex: egocentric and allocentric units were located on the same electrodes and cortical depths in the primary and posterior regions of auditory cortex in which we recorded. However, the low density of electrodes in our arrays, and their placement over a low-frequency border, prevented us from mapping the precise tonotopic boundaries necessary to attribute units to specific cortical subfields [41]. Future use of denser sampling arrays may enable cortical mapping in behaving animals and thus precise localization of allocentric units. We targeted the low-frequency reversal between primary and posterior auditory cortex, as these areas are likely to be sensitive to inter-aural timing cues, and the animals involved in this work were also trained to discriminate non-spatial features of low-frequency sounds in another study [42]. Posterior regions may correspond to part of the “what” pathway in auditory processing, whereas the anterior ectosylvian gyrus may correspond to the “where” pathway in which spatial tuning is more extensive [43,44]. It is thus likely that spatial tuning to the coordinate frames represented in our population (in which only 49% of units were spatially sensitive) may be more ubiquitous in anterior regions of ferret auditory cortex. Indeed, given that sensorimotor and cross-modal coordinate frame transformations are a key feature of activity in parietal cortex [10], it is likely that allocentric representations exist beyond auditory cortex.

While the observation of allocentric receptive fields in tonotopic auditory cortex is novel, the existence of allocentric representations has been predicted by behavioral studies in humans [6,13]. Furthermore, coordinate transformations occur elsewhere in the auditory system [23,45], and behavioral movements can influence auditory subcortical and cortical processing [29,30]. Perhaps most importantly, vestibular signals are already integrated into auditory processing at the level of the cochlea nucleus [46], allowing the distinction between self and source motion [22]. Auditory-vestibular integration, together with visual, proprioceptive, and motor corollary discharge systems, provides a mechanism through which changes in head direction can partially offset changes in acoustic input during movement to create allocentric representations. At present, it is unclear whether allocentric representations require self-generated movement, and it will be interesting to test if world-centered tuning is present if head direction is systematically varied in stationary animals.

It is also unclear how head position is also integrated into auditory processing. Positional information within the medial temporal lobe (or its carnivore equivalent) is a leading candidate given the connections between entorhinal and auditory cortex [47,48]; however, the functional interactions between these areas and their potential contributions to allocentric processing have yet to be addressed. Another critical question relates to the visual (or other sensory) cues that animals may use to orient in the world and define allocentric representations. Given that head and place cells remap in different settings [49,50] and that auditory cortex receives somatosensory and visual information through multiple pathways [48,51], it will be interesting to determine if changes in visual/tactile environment affect tuning of allocentric receptive fields, and if so, what environmental features anchor auditory processing.

Variation in the animal’s head position with movement also allowed us to study the effects of head movement speed on auditory processing and spatial tuning. In contrast to other studies in freely behaving animals that reported movement-related suppression of activity [29–31], we found that neurons tended to fire more strongly when the head moved faster (Fig 12). One reason for this difference may lie in the behavior measured: Other investigators have covered a diverse range of actions including locomotion in which both the head and body move, and self-generated sounds are more likely. We only considered the head speed of an animal and did not track the body position that would distinguish head movements from locomotion (which was relatively limited given the size of the animal and the arena). It is thus likely that much of the variation in speed we observed was a product of head movement during foraging without locomotion and thus with relatively little self-generated sound. The behavior of our subjects may therefore present different requirements for auditory–motor integration that result in distinct neural effects.

We also observed that spatial modulation was also greater when the animal was moving faster, which may be consistent with the sharpening of tuning curves during behavioral engagement [15]. While sharpening of engagement-related spatial tuning was linked to a reduction in spiking responses at untuned locations, we observed nonsignificant decreases in peak and minimum firing rates (which together increased modulation depth), suggesting that the mechanisms underlying speed-related modulation of spatial tuning may be subtly different. At the acoustic level, faster movements provide larger dynamic cues [2,3] that improve sound localization abilities in humans [3,52,53] and may explain the increase in modulation depth of units at greater speeds observed here.

In summary, we recorded spatial tuning curves in auditory cortex of freely moving animals to resolve coordinate frame ambiguity. We demonstrated many egocentric units representing location relative to the head and a small number of allocentric units representing sound location relative to the world. We also studied the role of distance and speed in auditory cortical processing. Together, our findings illustrate that auditory cortical processing of sound space may extend to multiple coordinate frames and spatial dimensions such as azimuth and distance, as well as non-auditory variables such as speed of movement.

## Methods

### Ethics statement

All experimental procedures were approved by local ethical review committees (Animal Welfare and Ethical Review Board) at University College London and The Royal Veterinary College, University of London and performed under license from the UK Home Office (Project License 70/7267) and in accordance with the Animals (Scientific Procedures) Act 1986.

### Simulated spatial receptive fields

#### Egocentric

Egocentric tuning described the relationship between spike probability (*P*) and sound source angle relative to the midline of the subject’s head (*θ*_*HS*_) and was simulated in Matlab (MathWorks) using a Gaussian function:

$$P\left(\theta \right)=ae^{-\frac{{\left(\theta_{HS}-b\right)}^{2}}{2c^{2}}}$$ (1)

In the example shown in Fig 1a, parameters (a = 1.044, b = 0°, and c = 75.7°) were determined by manual fitting to find values for which egocentric and allocentric tuning matched qualitatively. The theta domain was between ±180° binned at 1° intervals, and distance of sound sources was not included in the simulation.

#### Allocentric

Allocentric tuning describes the relationship between neural output (reported here as spike probability; *P*) and sound source position within the world measured in Cartesian (*x*, *y*) coordinates. Spatial tuning was simulated as the dot product of spike probability vectors returned from functions defined separately for positions on the *x*- and *y*-axes:

P(x,y)=f(x)⋅f(y) (2)

In Fig 1b, logistic probability functions were used for both dimensions:

$$f\left(x\right)=\;\;\frac{e^{-\frac{\left(x-\mu \right)}{s}}}{s{\left(1+e^{-\frac{\left(x-\mu \right)}{s}}\right)}^{2}}$$ (3)

$$f\left(y\right)=\;\;\frac{e^{-\frac{\left(y-\mu \right)}{s}}}{s{\left(1+e^{-\frac{\left(y-\mu \right)}{s}}\right)}^{2}}$$ (4)

With μ = 1,000 mm and s = 400 mm for the *x*-axis, and μ = 0 mm and s = 1,000 mm for the *y*-axis. For both axes, spike probability vectors were generated for domains between ±1,500 mm binned at 2-mm intervals.

#### Stimulus presentation, head pose, and movement

The position and orientation of the subject’s head within the world was described as a coordinate frame transform composed of a translation vector between origins (indicating the head position) and a rotation matrix between axes (indicating the head direction). Stimuli were presented on each time-step of the simulation from each speaker in a ring at 10° intervals, 1,000 mm from the origin of the world coordinate frame. As the simulation was deterministic, each stimulus was presented to static simulations only once to calculate the response. When simulating motion, a “pirouette” trajectory was constructed in which the subject’s head translated on a circular trajectory (radius = 50 mm; angular speed = 30° per time-step) while simultaneously rotating (angular speed = 10° per time-step) for 7,200 stimulus presentations (S2 Video). For each stimulus presentation, the stimulus angle was calculated relative to both the midline of the head and the vertical axis of the arena (S11 Fig). Simulation responses were quantized in 1° bins.

### Animals

Subjects were 5 pigmented female ferrets (1–5 years old) trained in a variety of psychophysical tasks that did not involve the stimuli presented or the experimental chamber used in the current study. Each ferret was chronically implanted with Warp-16 microdrives (Neuralynx, MT), housing 16 independently moveable tungsten microelectrodes (WPI Inc., FL) positioned over middle and posterior fields of left or right auditory cortex. Details of the surgical procedures for implantation and confirmation of electrode position are described elsewhere [54]. The weight and water consumption of all animals were measured throughout the experiment. Regular otoscopic examinations were performed to ensure the cleanliness and health of ferrets’ ears.

Subjects were water-restricted prior to testing, during which time they explored the experimental arena to find freely available sources of water. On each day of testing, subjects received a minimum of 60 ml/kg of water either during testing or supplemented as a wet mash made from water and ground high-protein pellets. Subjects were tested in morning and afternoon sessions on each day for up to 3 days in a week (i.e., a maximum of 6 consecutive testing sessions); on the remaining weekdays, subjects obtained water in performance of other psychophysical tasks. Test sessions lasted between 10 minutes and 50 minutes and were ended when the animal lost interest in exploring the arena.

### Experimental design and stimuli

In each test session, a ferret was placed within a D-shaped arena (Fig 2a, rectangular section: 35 cm x 30 cm [width x length]; semi-circular section: 17.5 cm radius; 50 cm tall) with 7 speakers positioned at 30° intervals, 26 cm away from a central pillar from which the animal could find water. The periphery of the circular half of the arena was also fitted with spouts from which water could be obtained. Animals were encouraged either to explore the arena by delivery of water at all spouts, or to hold their head at the center spout by restricted water delivery at this location. The arena and speakers were housed within a sound-attenuating chamber lined with 45-mm sound-absorbing foam.

During exploration (*n* = 57 test sessions), clicks were presented from each speaker with random inter-stimulus intervals (250–500 ms). The instantaneous energy of clicks minimized dynamic cues, simplifying neural analysis and comparisons with other work on spatial encoding. Clicks were presented at 60 dB SPL when measured from the center of the arena using a measuring amplifier (Bruel & Kjaer 2636). However, because sound level varied across the arena, we roved sound levels over a ±6 dB range to reduce changes in level arising from differences in position of the head within the sound field. The frequency response of each speaker (Visaton SC 5.9) was measured using golay codes [55] and compensated for, to produce a flat spectral output between 20 Hz and 20 kHz. Stimulus location and water delivery were independent and subjects were not required to attend to stimuli in order to find water rewards. To avoid characterizing neural responses to the sound of solenoid control signals, stimulus presentation and water reward were delivered in separate, alternating time windows; water was delivered in a short period of 1 to 2 seconds when each solenoid was rapidly opened (100 ms duration) with a 10-second interval between delivery windows in which click stimuli were presented. Sessions typically lasted approximately 15–20 minutes (median = 16.5 minutes; range = 6.15–48.0 minutes) in which several thousand stimuli could be presented (median = 1984; range = 304–3937).

### Head tracking

During exploration of the experimental arena, the animal’s head position and orientation were tracked using 2 LEDs (red and green) placed along the midline of the head and recorded using a RV2 video acquisition system (TDT) sampling at 30 frames per second and synchronized with the electrophysiology recording hardware. For each video frame, the red and green LED positions were identified in Matlab from a weighted image in which the channel color of the target LED was positively weighted and all other channels negatively weighted. Each LED position was then taken as the center of the largest cluster of pixels containing the maximum weighted value. To maximize the frame rate of the camera, we recorded with a low-exposure time (10–20 ms). Lower exposure also improved LED identification by reducing signal intensity in the background of each frame.

In cases in which a LED went out of view of the camera (usually due to the roll or pitch of the head, or the recording cables obscuring the LED), the maximum weighted value identified as the LED would be a random point within the arena, resulting either from a weak reflection or image noise. To remove such data, we set a minimum-intensity threshold based on the distribution of maximum values in weighted images across all frames. In cases in which the LED intensity failed to match the specified threshold, the LED position was noted as missing. To compensate for missing data, we estimated LED positions across runs of up to a maximum of 10 frames (333 ms) using spline interpolation. Longer runs of missing data were discarded.

We then mapped each LED position in the image (*M*) into the behavioral arena to give the new position *N* using the transformation:

N= T+RM (5)

Where *T* is the translation between the origin of the image coordinate frame (i.e., pixel [0,0]) and the origin of the arena coordinate frame (the center of the arena). And, *R* is the three-dimensional rotation matrix describing the rotation between the arena and image coordinate frames.*T* was obtained by manually identifying the pixel closest to the center of the arena (i.e., the equidistant point between all speakers) in a calibration image captured at the start of each test session. *R* was estimated from singular value decomposition using the position and distance between known points in the arena (also identified manually from each calibration image). Here, we estimated a 3D rotation matrix to take into account the position of the camera relative to the arena (i.e., above the arena rather than below). All coordinate frames were represented using the right-hand rule (i.e., positive values for counter-clockwise rotation about the *z*-axis) to ensure consistency with the *atan2* function.

The animal’s head position $\left(\vec{AH}\right)$ was then calculated as the midpoint between the LEDs within the arena and was used to define the origin of the head-centered coordinate frame (S11 Fig). The animal’s head direction (*θ*_*HA*_) was calculated from the two argument arctangent function (atan2) of the vector between LEDs that defined the midline (*y*-axis) of the head-centered coordinate frame $\left(\hat{j_{H}}\right)$. The *z*-axis was undefined by the tracking system, as we only measured 2 points (red and green LEDs) with a single camera; this led to ambiguity about the pitch and roll of the head. To compensate for this deficiency, we assumed that when LEDs were visible, the *xy* plane of the head always matched the plane of the arena floor and that the *z*-axis of the head was orthogonal to this plane and oriented towards the camera. Such assumptions are justified by the properties of the tracking system—as the head rolls or pitches away from the assumed conditions, it becomes impossible to identify both LEDs within the image due to the limited angular range of the each diode. Therefore, tracking was impossible (in which case data was discarded) in the same conditions in which our assumptions became untenable.

By using the frame times recorded on the device, it was possible to create a time series of head position and direction within the arena that could be compared to the spiking pattern of neurons. We used the inter-frame interval and change in position of the head origin, smoothed with a 9-point Hann window to calculate the speed of head movement.

### Neural recording

Neural activity in auditory cortex was recorded continuously throughout exploration. On each electrode, voltage traces were recorded using TDT System III hardware (RX8 and RZ2) and OpenEx software (Tucker-Davis Technologies, Alachua, FL) with a sample rate of 25 kHz. For extraction of action potentials, data were band-pass filtered between 300 Hz and 5,000 Hz, and motion artifacts were removed using a decorrelation procedure applied to all voltage traces recorded from the same microdrive in a given session [56]. For each channel within the array, we identified candidate events as those with amplitudes between −2.5 and −6 times the RMS value of the voltage trace and defined waveforms of events using a 32-sample window centered on threshold crossings. Waveforms were then interpolated (128 points) and candidate events combined across sessions within a test run for spike sorting. Waveforms were sorted using MClust (A.D. Redish, University of Minnesota, http://redishlab.neuroscience.umn.edu/MClust/) so that candidate events were assigned to either single unit, multi-unit clusters, or residual hash clusters. Single units were defined as those with less than 1% of inter-spike intervals shorter than 1 millisecond. In total, 331 units were recorded, including 116 single units (35.1%).

### Tracking unit identity across recording sessions

Through the experiment, electrodes were descended progressively deeper into cortex at intervals of 50–100 μm to ensure sampling of different neural populations. At most recording sites, we tested animals on multiple sessions (1–6 sessions) across several (1–3) consecutive days. Conducting test sessions over multiple days makes possible the recording of different units at a single recording site over time (i.e., through electrode drift, gliosis, etc.). To constrain our analysis to units with a consistent identity, we tracked the waveform of recorded units across sessions within a test run. Our rationale was that a unit should have a constant waveform shape across test sessions, and any differences in waveform shape should be small relative to differences in the waveforms of units measured on other electrodes or at other depths by the same electrode. Thus, for one test session at a given recording site, we calculated the Euclidean distance matrix between the mean waveform recorded on that session (W_Test_) and the mean waveform recorded on each additional session at the same recording site (D_Test_). We also calculated the distances between W_Test_ and the mean waveform recorded for every session at different recording sites (D_Control_). D_Control_ provided null distributions for waveform distances between pairs of neurons known to have separate identities (due to the spatial separation between electrodes at recording sites [>50 μm in depth, >500 μm laterally]). For a given waveform, we then calculated the statistical probability of observing distances between test waveform and waveforms **at the same recording site**, given the distribution of distances between test waveforms and waveforms **at other recording sites**. For waveforms exceeding statistical significance (*t* test; *p* < 0.05, Bonferroni corrected for the number of sessions conducted at the recording site), we concluded that the same neuron or neuronal population was recorded.

For runs of test sessions, we took the longest continuous run for which waveform distances were significantly smaller than expected by chance. The majority of units tested more than once could be tracked over all sessions tested (72.4%: 126/174 units), although the number of neurons tracked fell off with time.

### Data analysis

During exploration, we characterized sound-evoked responses from auditory cortical units. Each click stimulus and the concomitant neural response could be related to controlled variables determined by the experimental design and measured variables observed from head tracking. Controlled variables were the position of the sound source relative to the arena and sound source level in dB SPL, whereas measured variables were the position and direction of the head relative to the arena, as well as head speed. Controlled and measured variables were combined to determine several experimental parameters: Stimulus position relative to the head was calculated as the vector $\vec{HS}$:

$$\vec{HS}=\vec{AS}-\vec{AH}$$ (6)

Where $\vec{AH}$ is the vector from arena origin to head origin and $\vec{AS}$ is the vector from arena origin to the sound source. Stimulus angle relative to the head (*θ*_*HS*_) was calculated by subtracting head direction in the arena (*θ*_*HA*_) from the stimulus angle relative to the origin of the head coordinate frame:

$$\theta_{HS}=\text{ atan}2\left({\vec{HS}}_{y},{\vec{HS}}_{x}\right)-\;\theta_{HA}$$ (7)

The distance between head and stimulus was calculated as the magnitude of $\vec{HS}$.

To calculate sound level at the head, sound pressure at the head was calculated by multiplying the pressure measured at the arena origin during calibration (*p*_*A*_) by the ratio of the distances from arena origin to speaker $\left(\left|\vec{AS}\right|\right)$ and from head to speaker $\left(\left|\vec{HS}\right|\right)$:

$$p_{H}=\;p_{A}\cdot \frac{\left|\vec{AS}\right|}{\left|\vec{HS}\right|}$$ (8)

Where P_H_ and P_A_ are sound pressures at the head and center of the arena expressed in pascals, and sound level is expressed in dB SPL:

$$L=20\;\cdot log_{10}\left(\frac{\tilde{p}}{2\times 10^{-5}}\right)$$ (9)

Sound level was calibrated to 60 dB SPL (0.02 Pa) at the center of the arena.

For each variable, we calculated the value at the time of stimulus presentation (i.e., with a lag of 0 ms) and contrasted these values with the spiking responses of neurons. To study encoding of stimulus features (both measured and control variables) by neurons, single trial responses of individual units were summarized as the mean firing rate 0–50 ms after stimulus onset. This window was sufficiently long to characterize the response of units (S5 and S7 Figs) but also short enough that changes in head direction and position during the analysis window were small (S3 Fig). Sound-responsive units (268/336) were first identified as those with evoked firing rates that differed significantly from background activity measured in the 50 ms before stimulus presentation (GLM analysis of deviance using Poisson distributions and log link function; *p* ≤ 0.05).

Spatially tuned units were then identified using sound-evoked responses collected with the animal at the center of the arena, with the head and world coordinate frames in approximate alignment. For a stimulus presentation to be included in this analysis, the animal’s head origin was required to be within 5 cm of a point 2.5 cm behind the arena center (S4 Fig). The 2.5-cm offset was applied to provide an approximate account for the distance between the animal’s snout and head center. Head direction was also required to be within ±15° of the midline of the arena (i.e., the line of symmetry of the arena, so that the animal was facing forward). Sound-evoked responses under these constraints were then fitted with a GLM (Poisson distribution; log link function) with sound source angle relative to the head binned in 30° intervals as predictor. To ensure adequate data for statistical testing, units were only assessed if responses were recorded for ≥5 stimulus presentations in each angular bin (186/268 units). Units for which sound source angle significantly reduced model deviance (χ^2^ distribution, *p* ≤ 0.05) were classed as spatially tuned (92/186 units). While this approach may not identify all spatially informative neurons (some of which may signal sound location by spike timing rather than rate [18,57] or that may be tuned only to sounds behind the head that were not sampled by speakers in the aligned condition), it identified a subpopulation of spatially sensitive units on which further analysis could be performed.

To calculate spatial tuning curves, analysis was expanded to include all head positions and directions recorded. To calculate world-based tuning curves, mean firing rate across trials (0–50 ms) was plotted for each sound source angle relative to the arena. For head-based tuning curves, sound source angle relative to the head was binned at 30° intervals and mean spike rate plotted as a function of the bin center angle. To study super-resolution tuning of egocentric units (Fig 5a and 5b), the bin width used to calculate curves was reduced to 20°, 10°, 5°, 2°, or 1°. To compare spatial tuning of egocentric units with other studies, we also calculated preferred location, modulation depth, tuning width, and equivalent rectangular receptive field (ERRF) width for spatial tuning curves calculated relative to the head across 360°, according to the methods used for awake cats [15,18]. For allocentric units, we calculated preferred location and modulation depth for across sound location in the world.

### Modulation depth analysis

For each unit, we calculated the depth of spatial modulation for tuning curves in each coordinate frame. Unless otherwise stated, modulation depth (MD) was calculated as:

$$MD=\;\frac{\text{max}\left(x\right)-\text{ min}\left(x\right)}{\text{max}\left(x\right)}\;\times \;100$$ (10)

Where *x* is the vector of firing rates in response to sounds located in each 30° bin between ±90° either of the world or head coordinate frame.

Modulation depth could also be calculated for simulated neurons using the same equation but with *x* being a vector of spike probabilities. This approach allowed us to calculate modulation depth for simulated allocentric and egocentric units when presented with sounds during observed animal movement (Fig 3). In simulations, modulation depth could be calculated in head and world coordinate frames that were either relevant or irrelevant for neural activity, depending on whether the simulation was allocentric or egocentric. We termed modulation depth in the irrelevant coordinate frame **residual modulation** when expressed as a ratio of modulation in the represented coordinate frame:

$$Residual\;Modulation\;=\;\frac{MD_{Irrelevant}}{MD_{Relevant}}$$ (11)

For allocentric simulations, the world was relevant and the head was irrelevant; whereas, for egocentric simulations, the head was relevant and the world was irrelevant.

For each test session in which we observed animal behavior, we compared the relationship between the residual modulation calculated during simulations of both allocentric and egocentric units, with the standard deviation of the head directions (σ, Fig 3). We fitted a linear regression model to this relationship that was subsequently used to test if observed modulation depth values of real units were significantly greater than the residual modulation expected from the animal’s behavior. The linear regression model was fitted using the **fitlm** function in Matlab (R2015a). For each observed unit, we measured the standard deviation of head angles during neural testing (σ) and, together with the regression models, predicted the 95% or 99.95% (post-Bonferroni correction for 92 units) confidence interval of residual modulation values in the head and in the world coordinate frame. Prediction was performed in Matlab using the **predict** function with the most conservative options selected (simultaneous confidence bounds and prediction for new observations rather than fitted mean values) to give the widest confidence intervals and thus minimize the probability of false positives. If the observed modulation depth of a unit in a particular coordinate frame exceeded the upper bound of the confidence interval for that frame, we identified it as significantly modulated.

### GLMs

To compare the relationship between single trial firing rates and sound source angles in the head and world coordinate frames, we fitted the average firing rate on each trial (0–50 ms) with a generalized linear regression model (Matlab, **fitglm** function: Poisson distribution, log link function). For both sound source angles relative to the head and relative to the world, we measured the deviance of models fitted separately with each parameter (D_Test_). The AIC [24] was used to compare test models and distinguish allocentric and egocentric units as those for which sound source angle relative to the world or head, respectively, provided the best model. For all but 1 unit that was excluded from further analysis, either sound source angle relative to the world or head improved model fit compared to a constant model (analysis of deviance; Bonferroni correction for 2 comparisons; *p* < 0.05).

To visualize GLM performance (Fig 6), we calculated **model fit** for each unit and coordinate frame as:

$$Model\;Fit=\;\frac{D_{Const}-D_{Test}}{D_{Const}-D_{Full}}$$ (12)

Where D_const_ was the deviance resulting from a constant model, and D_Full_ was the deviance resulting from a full linear model that included both sound source angle relative to the head and relative to the world. We compared the model fit for data obtained when the head and world coordinate frames were free to vary and when we restricted data to cases when the head and world coordinate frames were aligned (see above). We also repeated our analysis but with speaker identity or head direction information randomly shuffled between stimulus presentations prior to calculation of spatial tuning curves. Shuffling was repeated 1,000 times and, across shuffles, we calculated the median model fit in head and world coordinate frames. Here we used the median rather than mean of the permuted values across shuffles, which were not always normally distributed. To test the effect of shuffle on model fit of all units, we performed a 2-way anova on change in model fit with shuffle, with coordinate frame (head/world) and unit class (egocentric/allocentric). Post hoc tests were conducted on change in model fit versus 0 (*t* test) with Bonferroni correction for multiple comparisons.

For each analysis in which we calculated model fit, we also calculated **model preference** as:

$$Model\;Preference=\;Model\;Fit_{Head}-\;Model\;Fit_{World}$$ (13)

Model preference could thus vary between −100% (better fit for neural data based on sound angle in the world) and +100% (better fit for neural data based on sound angle relative to the head).

For time-based comparison of model performance, we reduced the time over which firing rates were considered (from 50 ms to 20 ms) and repeated the analysis with a window offset by −60 ms to 90 ms after stimulus presentation that moved with a 2-ms interval. Model fit and preference values for allocentric and egocentric units were compared across time using a nonparametric cluster-based statistical test [26], implemented in Matlab through the FieldTrip toolbox [58].

## Supporting information

S1 Fig Allocentric tuning can generate a wide variety of tuning curve shapes and tuned locations. **a**, Variation in tuning shape generated by allocentric simulations tuned to different regions of space and implemented using different spike probability distributions: Simulations (left to right) based on logistic (red), Gaussian (black), Laplace (blue) and uniform (grey) probability density functions. **b**, Shifts in tuning simulated by varying world-based position of peak spike probability. Plots generated from logistic probability density functions. Data available at https://doi.org/10.6084/m9.figshare.4955390.v1.
(TIF)

S2 Fig Electrode positions in auditory cortex. **a**, Electrode positions in two representative animals in auditory cortex. Green and black indicate electrodes within or outside (excluded from analysis) Auditory Cortex respectively. Labels show suprasylvian sulcus (sss) and pseudosylvian sulcus (pss). **b**, Cresyl-violet stained electrode tracks.
(TIF)

S3 Fig Head movement during neural analysis window. **a**, Time-displacement plots showing the change in head position across space (black), in each axis of the arena (red and blue) and change in head direction (green) during the 50 millisecond window over which spike rates were analysed. Data shown as mean ± s.e.m with grey lines showing behavior of every session (n = 57). **b**, Box plots of median displacement 50 milliseconds after click onset in each analysis. **c-d** Same as a-b but plotted for 2 milliseconds after click onset. Data available at https://figshare.com/articles/S3_Fig/4955393.
(TIF)

S4 Fig Control data showing spatial tuning when head and world coordinate frames aligned. **a**, Distribution of head position (left), head directions (top right) and head speeds (bottom right, n = 57 test sessions, mean ± s.e.m.) for control data which has been filtered for positions at the center of the arena with the head facing forward. **b**, Spatial tuning functions calculated for sound source angle relative to the head and world aligned for filtered data (inset shows tuning for one unit, n = 275 stimuli). R^2^ indicates correlation coefficient between functions calculated for each coordinate frame. Histogram shows distribution of correlation coefficients across all spatially tuned units. **c**, Population tuning functions expressed in head centered coordinate frame illustrating contralateral tuning bias for neurons recorded in left and right hemispheres (n = 64 and 28 respectively). Data available at https://doi.org/10.6084/m9.figshare.4955399.v1.
(TIF)

S5 Fig Example egocentric raster plot. Raster plots showing spiking responses of an example egocentric unit (Fig 4b in the main manuscript). Data available at https://doi.org/10.6084/m9.figshare.4955402.v1.
(TIF)

S6 Fig Additional examples of egocentric units. Two additional example egocentric units in which spatial receptive fields are tuned to sound source location in the head coordinate frame. Data shown as in Fig 4c of main text with line plots showing mean ± s.e.m. Data available at https://doi.org/10.6084/m9.figshare.4955417.v1.
(TIF)

S7 Fig Example allocentric raster plot. Raster plots showing spiking responses of an example allocentric unit (Fig 4c in the main manuscript). https://doi.org/10.6084/m9.figshare.4955405.v1.
(TIF)

S8 Fig Additional examples of allocentric units. Two additional example allocentric units in which spatial receptive fields are tuned to sound source location in the world coordinate frame. Data shown as in Fig 4 of main text with line plots showing mean ± s.e.m. Data available at https://doi.org/10.6084/m9.figshare.4955414.v1.
(TIF)

S9 Fig Measuring super-resolution spatial tuning. Spatial tuning curves for three examples of egocentric units for which tuning was observed in 360° around the head and with resolution greater than the interval between speakers (30°). Typically units showed reliable tuning around the head at resolutions as low as 5°, equivalent to using a speaker ring with 72 speakers positioned at equal intervals around the animal’s head in the azimuthal plane. As we used only 7 speakers over a range of 180°, this reflects an order of magnitude (x 10) increase in spatial resolution. Data shown as mean ± s.e.m firing rate in Cartesian or polar coordinates. Scale bars indicate firing rates of 20 Hz. Data available at https://doi.org/10.6084/m9.figshare.4955408.v1.
(TIF)

S10 Fig Temporal differences when coordinate frames were aligned. Population distinctions when head and world coordinate frames are aligned (in contrast to the main text where frames were free to vary). **a**, Model fit for predicting neural activity from sound angles relative to the head. **b**, Model fit for predicting neural activity from sound angles in the world. **c**, Model preference. Data shown as mean ± s.e.m. for egocentric (blue) and allocentric (red) populations. Populations did not differ significantly at any time point (cluster based paired t-test, p< 0.05). Data available at https://doi.org/10.6084/m9.figshare.4955411.v1.
(TIF)

S11 Fig Geometric definitions. (TIF)

S1 Video Low exposure video example of LEDs during motion tracking (left) that allows projection of head position and direction within the experimental arena (right). (MP4)

S2 Video Pirouette trajectory used to obtain a uniform distribution of head directions in simulations. (MP4)

## Abbreviations

AIC: Akaike information criterion
CF: coordinate frame
CI: confidence interval
dB SPL: decibel sound pressure level
EEG: electroencephalography
ERRF: equivalent rectangular receptive field
GLM: general linear model
ILD: inter-aural level difference
LED: light-emitting diode
