# Supplementary figures and images for: Egocentric and allocentric representations in auditory cortex
Source: PLoS Biol. 2017 Jun 15;15(6):e2001878. doi: 10.1371/journal.pbio.2001878 (PMC5472254; doi:10.1371/journal.pbio.2001878)

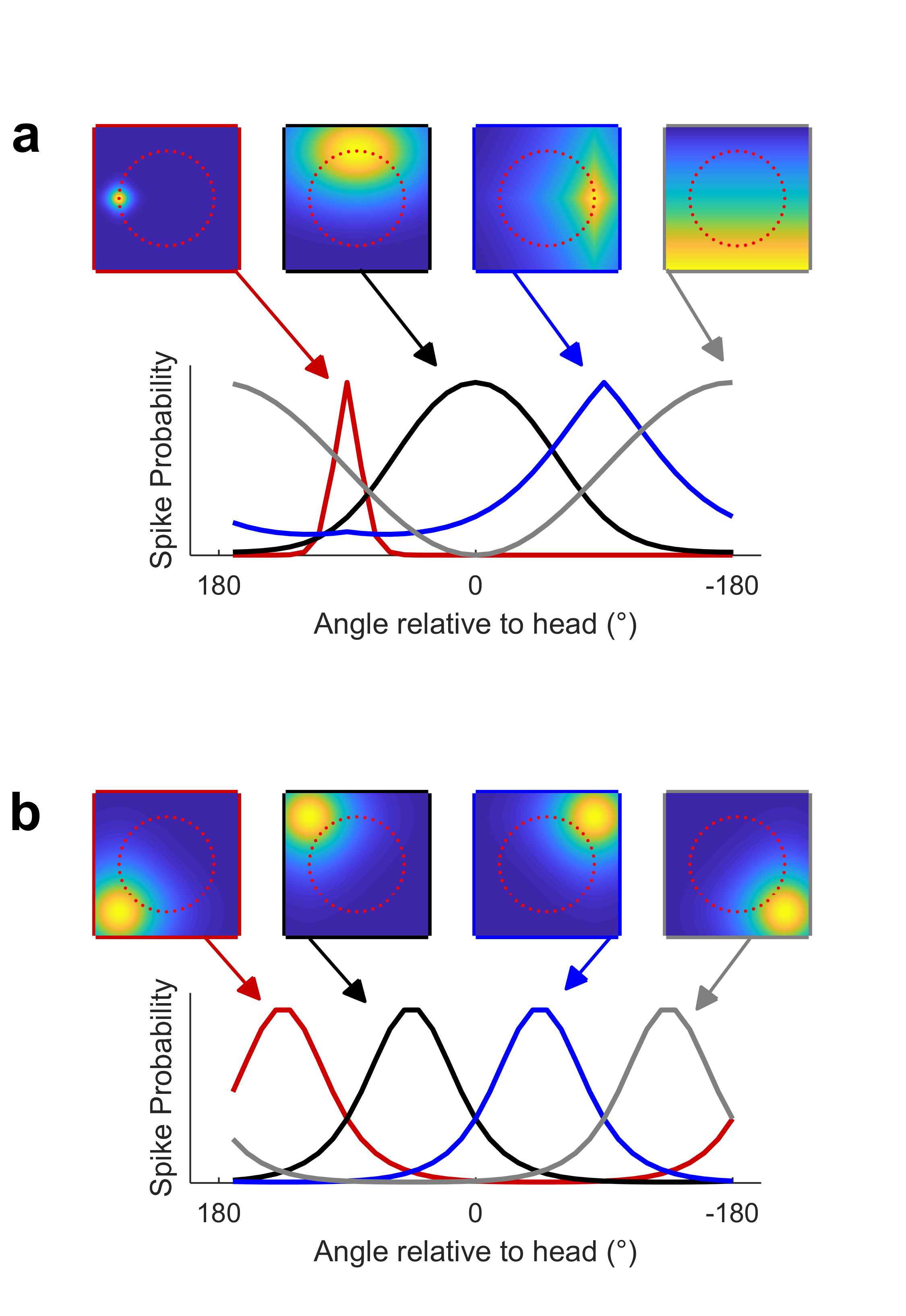

Supplement: S1 Fig — a, Variation in tuning shape generated by allocentric simulations tuned to different regions of space and implemented using different spike probability distributions: Simulations (left to right) based on logistic (red), Gaussian (black), Laplace (blue) and uniform (grey) probability density functions. b, Shifts in tuning simulated by varying world-based position of peak spike probability. Plots generated from logistic probability density functions. Data available at https://doi.org/10.6084/m9.figshare.4955390.v1. (TIF) [file pbio.2001878.s001.tif]

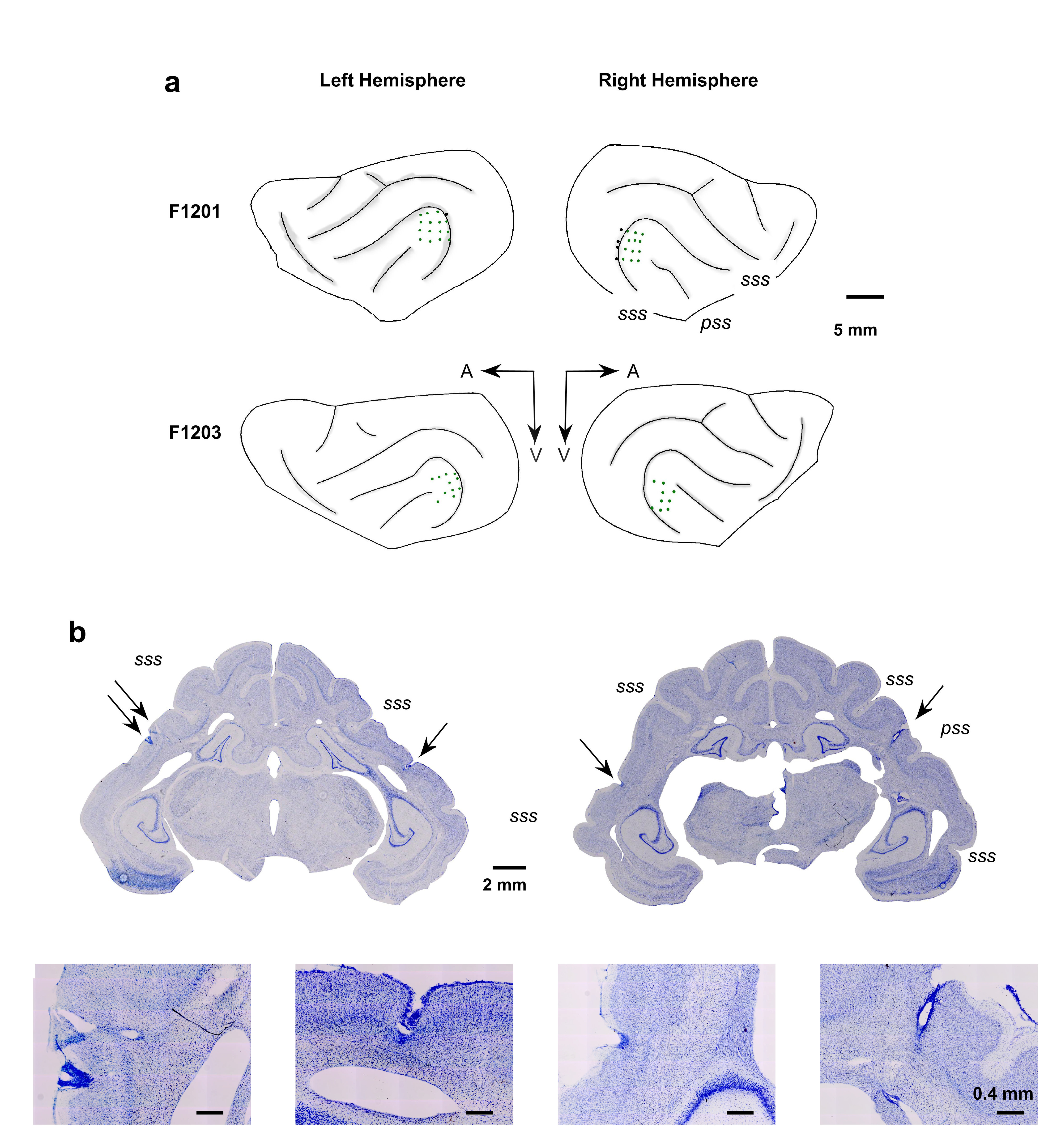

Supplement: S2 Fig — a, Electrode positions in two representative animals in auditory cortex. Green and black indicate electrodes within or outside (excluded from analysis) Auditory Cortex respectively. Labels show suprasylvian sulcus (sss) and pseudosylvian sulcus (pss). b, Cresyl-violet stained electrode tracks. (TIF) [file pbio.2001878.s002.tif]

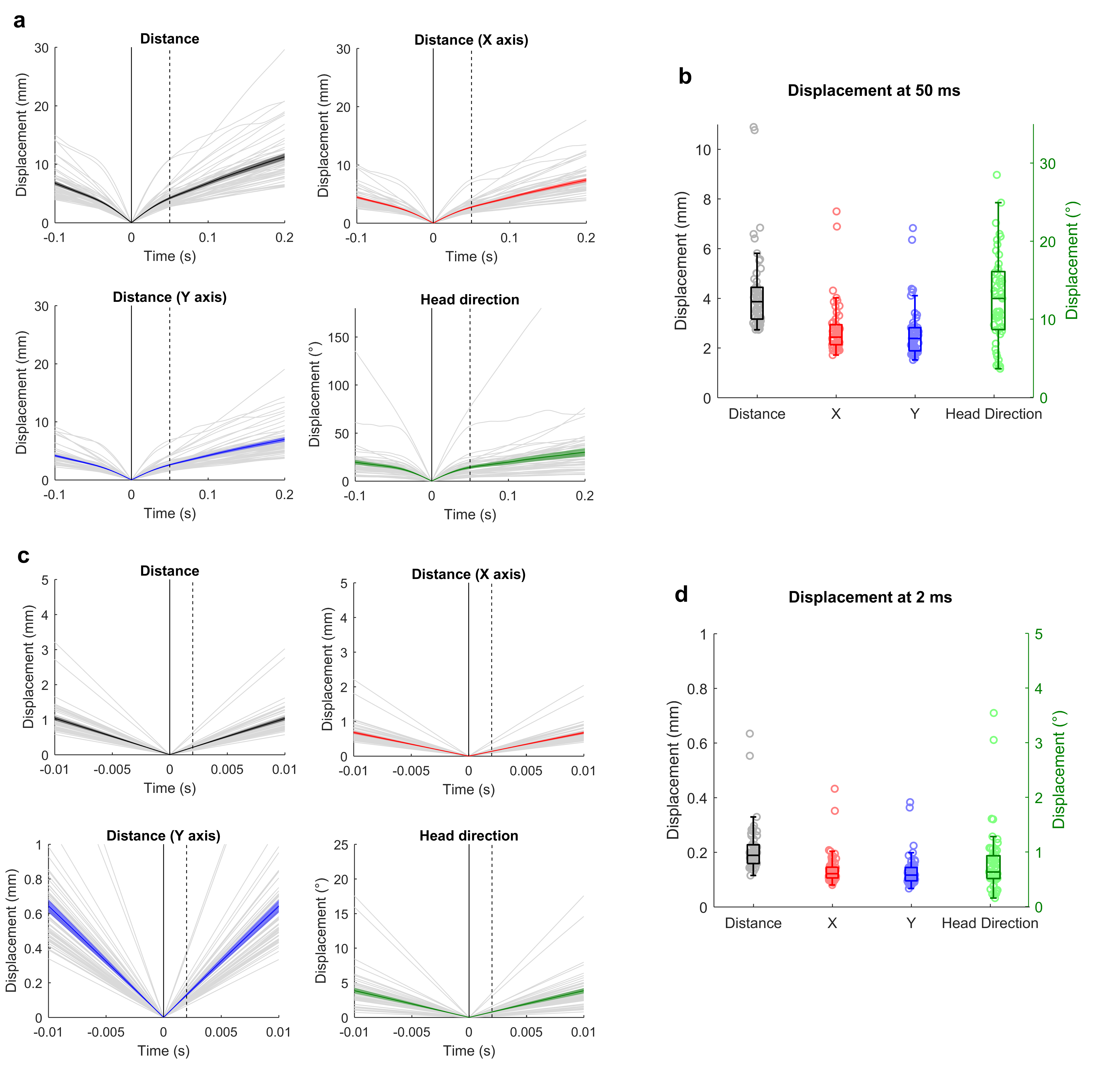

Supplement: S3 Fig — a, Time-displacement plots showing the change in head position across space (black), in each axis of the arena (red and blue) and change in head direction (green) during the 50 millisecond window over which spike rates were analysed. Data shown as mean ± s.e.m with grey lines showing behavior of every session (n = 57). b, Box plots of median displacement 50 milliseconds after click onset in each analysis. c-d Same as a-b but plotted for 2 milliseconds after click onset. Data available at https://figshare.com/articles/S3_Fig/4955393. (TIF) [file pbio.2001878.s003.tif]

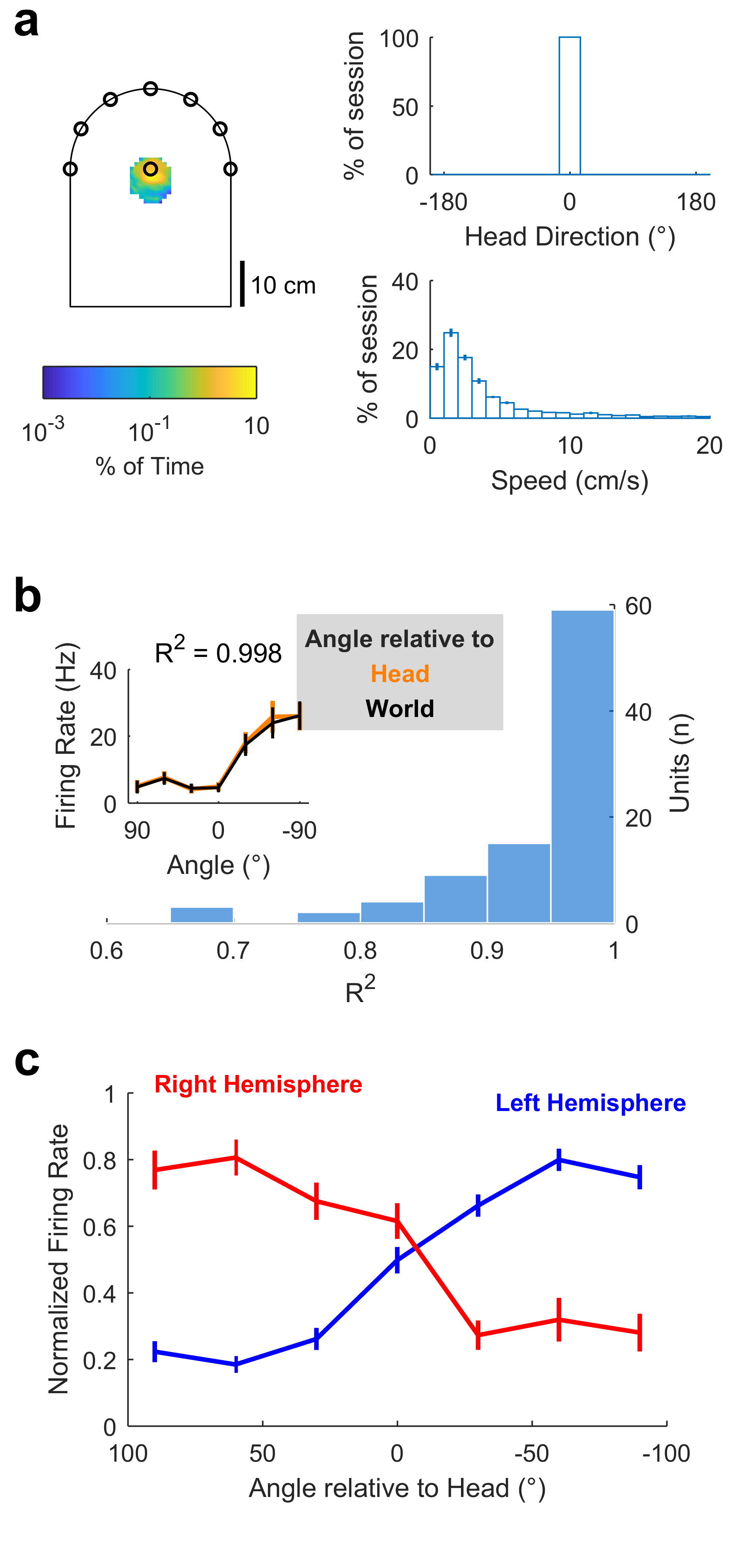

Supplement: S4 Fig — a, Distribution of head position (left), head directions (top right) and head speeds (bottom right, n = 57 test sessions, mean ± s.e.m.) for control data which has been filtered for positions at the center of the arena with the head facing forward. b, Spatial tuning functions calculated for sound source angle relative to the head and world aligned for filtered data (inset shows tuning for one unit, n = 275 stimuli). R2 indicates correlation coefficient between functions calculated for each coordinate frame. Histogram shows distribution of correlation coefficients across all spatially tuned units. c, Population tuning functions expressed in head centered coordinate frame illustrating contralateral tuning bias for neurons recorded in left and right hemispheres (n = 64 and 28 respectively). Data available at https://doi.org/10.6084/m9.figshare.4955399.v1. (TIF) [file pbio.2001878.s004.tif]

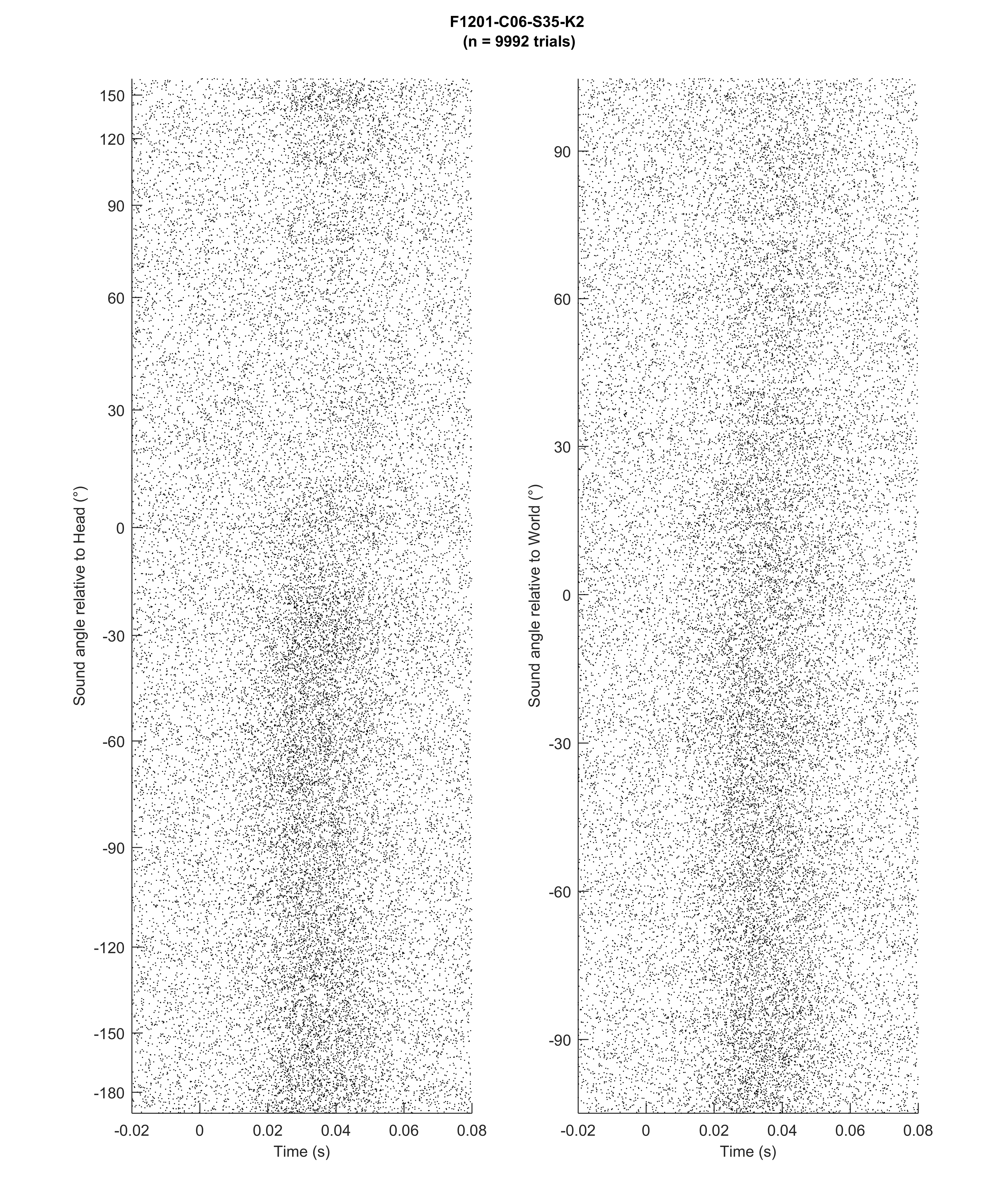

Supplement: S5 Fig — Raster plots showing spiking responses of an example egocentric unit (Fig 4b in the main manuscript). Data available at https://doi.org/10.6084/m9.figshare.4955402.v1. (TIF) [file pbio.2001878.s005.tif]

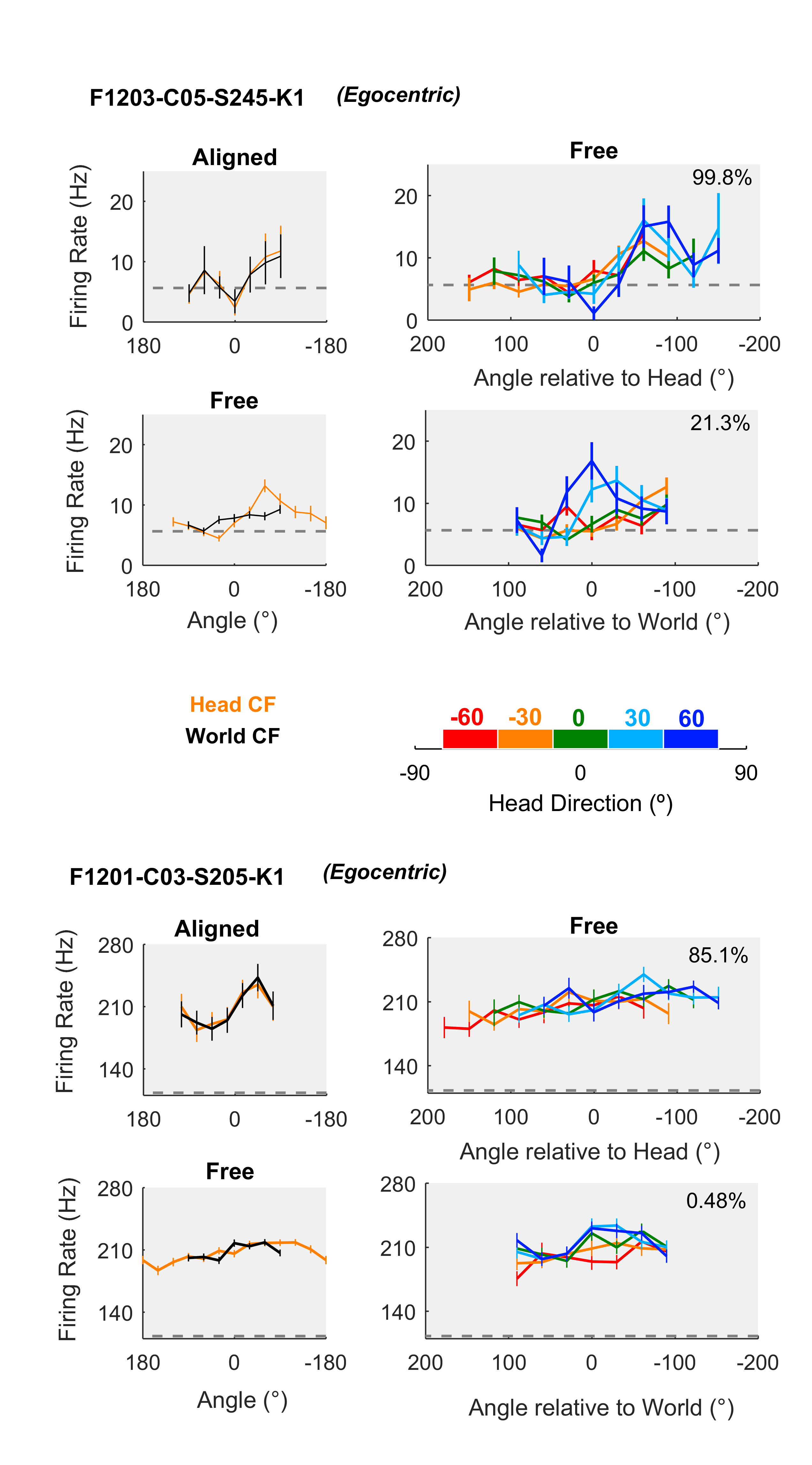

Supplement: S6 Fig — Two additional example egocentric units in which spatial receptive fields are tuned to sound source location in the head coordinate frame. Data shown as in Fig 4c of main text with line plots showing mean ± s.e.m. Data available at https://doi.org/10.6084/m9.figshare.4955417.v1. (TIF) [file pbio.2001878.s006.tif]

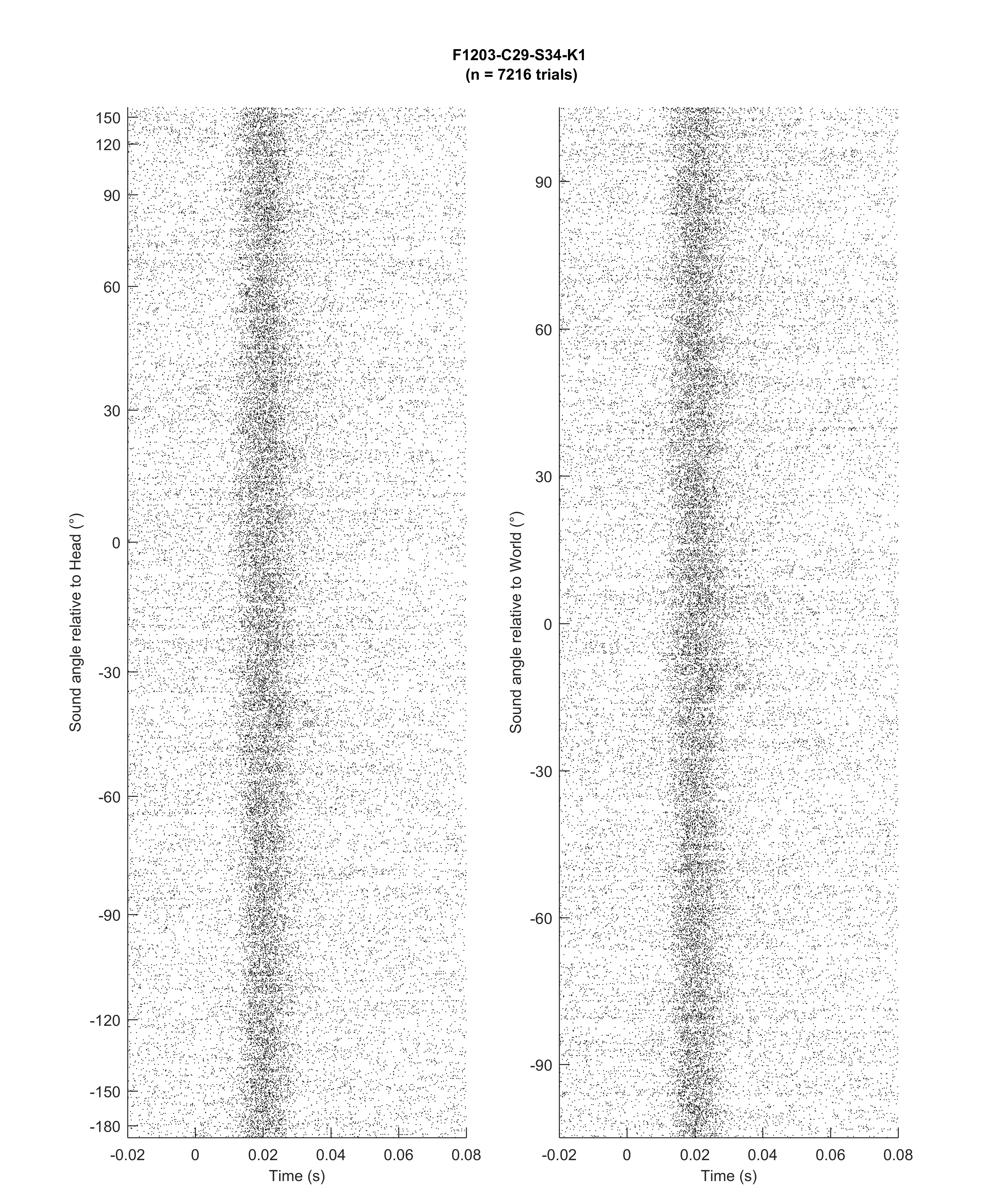

Supplement: S7 Fig — Raster plots showing spiking responses of an example allocentric unit (Fig 4c in the main manuscript). https://doi.org/10.6084/m9.figshare.4955405.v1. (TIF) [file pbio.2001878.s007.tif]

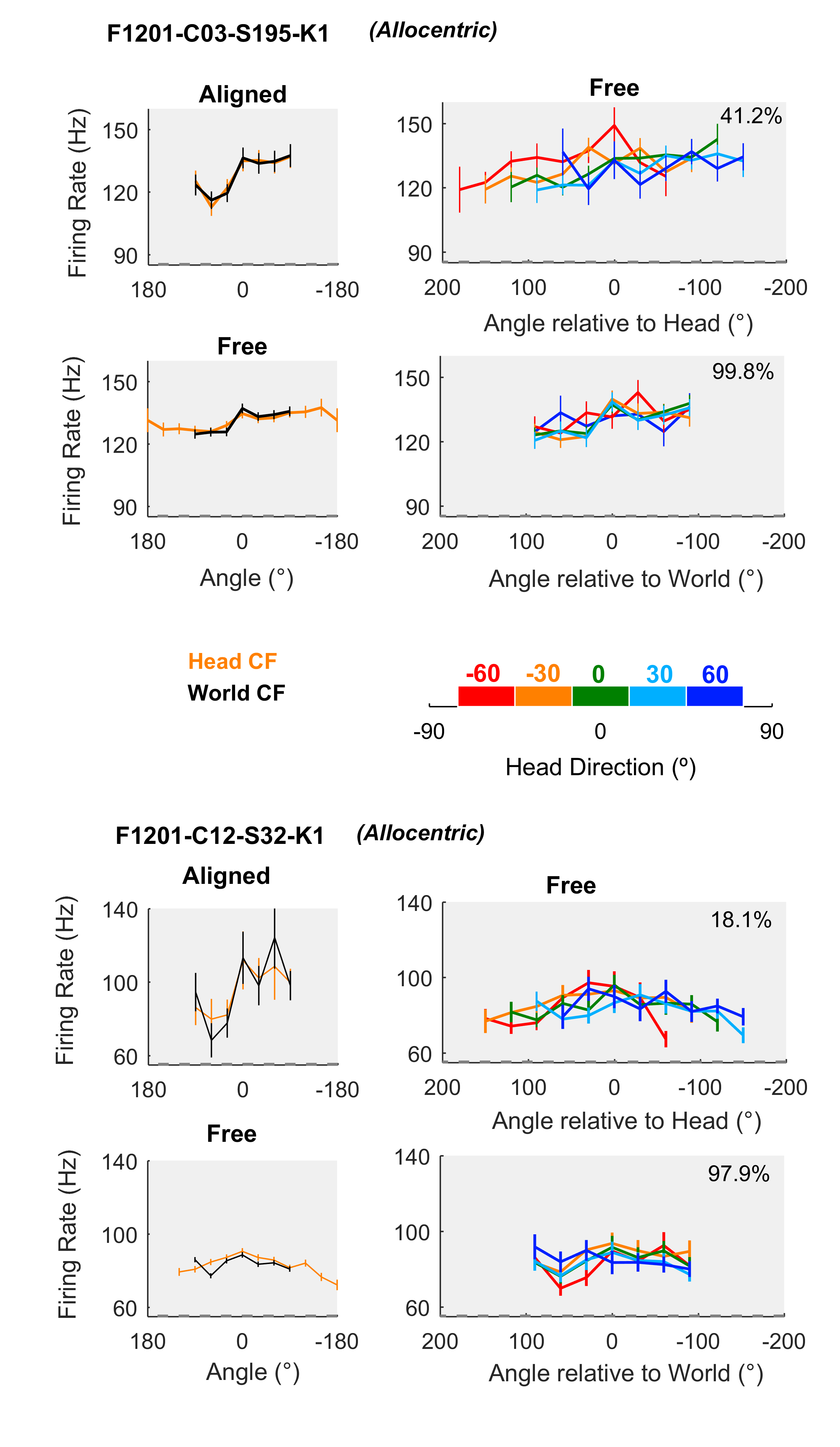

Supplement: S8 Fig — Two additional example allocentric units in which spatial receptive fields are tuned to sound source location in the world coordinate frame. Data shown as in Fig 4 of main text with line plots showing mean ± s.e.m. Data available at https://doi.org/10.6084/m9.figshare.4955414.v1. (TIF) [file pbio.2001878.s008.tif]

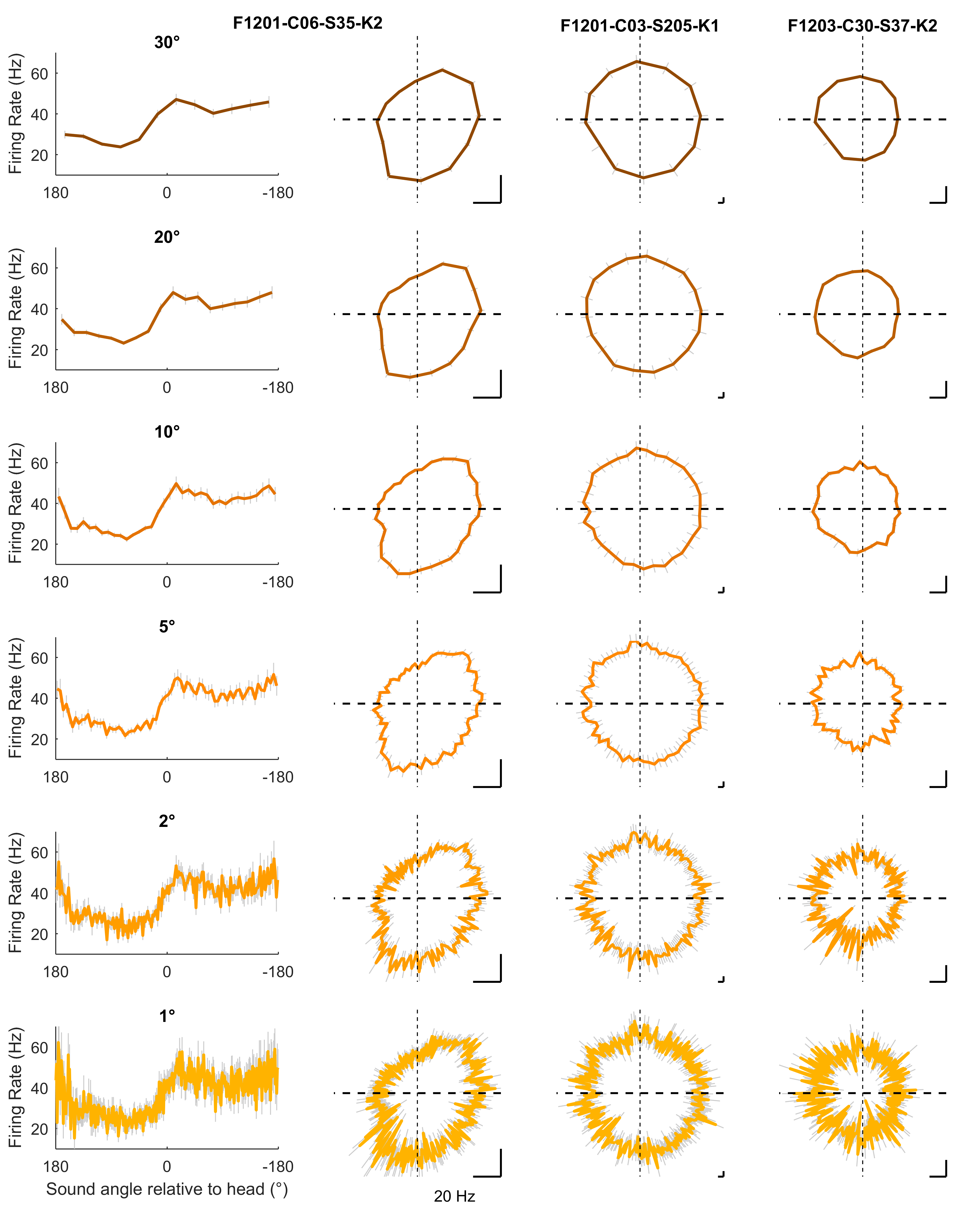

Supplement: S9 Fig — Spatial tuning curves for three examples of egocentric units for which tuning was observed in 360° around the head and with resolution greater than the interval between speakers (30°). Typically units showed reliable tuning around the head at resolutions as low as 5°, equivalent to using a speaker ring with 72 speakers positioned at equal intervals around the animal’s head in the azimuthal plane. As we used only 7 speakers over a range of 180°, this reflects an order of magnitude (x 10) increase in spatial resolution. Data shown as mean ± s.e.m firing rate in Cartesian or polar coordinates. Scale bars indicate firing rates of 20 Hz. Data available at https://doi.org/10.6084/m9.figshare.4955408.v1. (TIF) [file pbio.2001878.s009.tif]

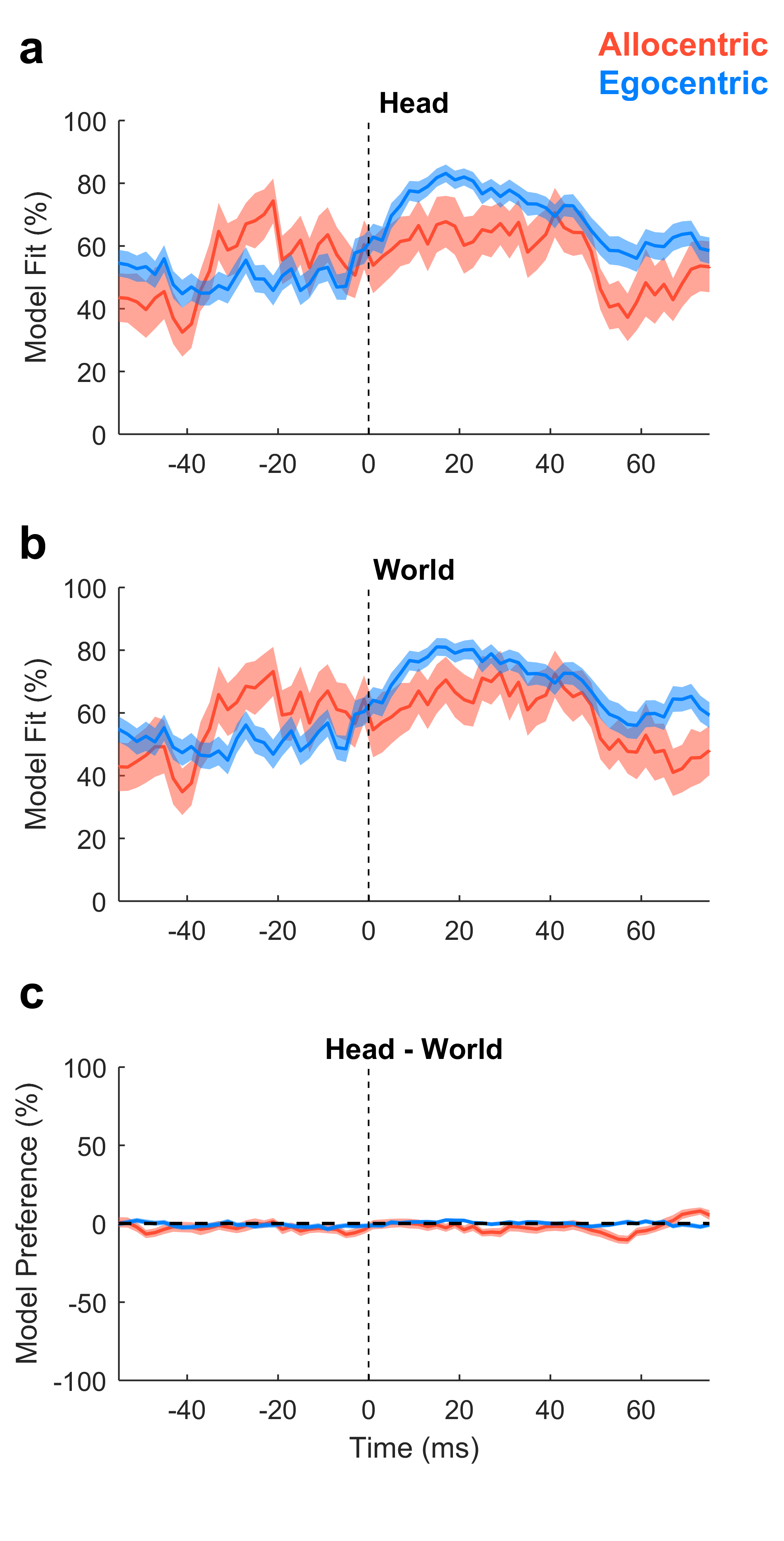

Supplement: S10 Fig — Population distinctions when head and world coordinate frames are aligned (in contrast to the main text where frames were free to vary). a, Model fit for predicting neural activity from sound angles relative to the head. b, Model fit for predicting neural activity from sound angles in the world. c, Model preference. Data shown as mean ± s.e.m. for egocentric (blue) and allocentric (red) populations. Populations did not differ significantly at any time point (cluster based paired t-test, p< 0.05). Data available at https://doi.org/10.6084/m9.figshare.4955411.v1. (TIF) [file pbio.2001878.s010.tif]

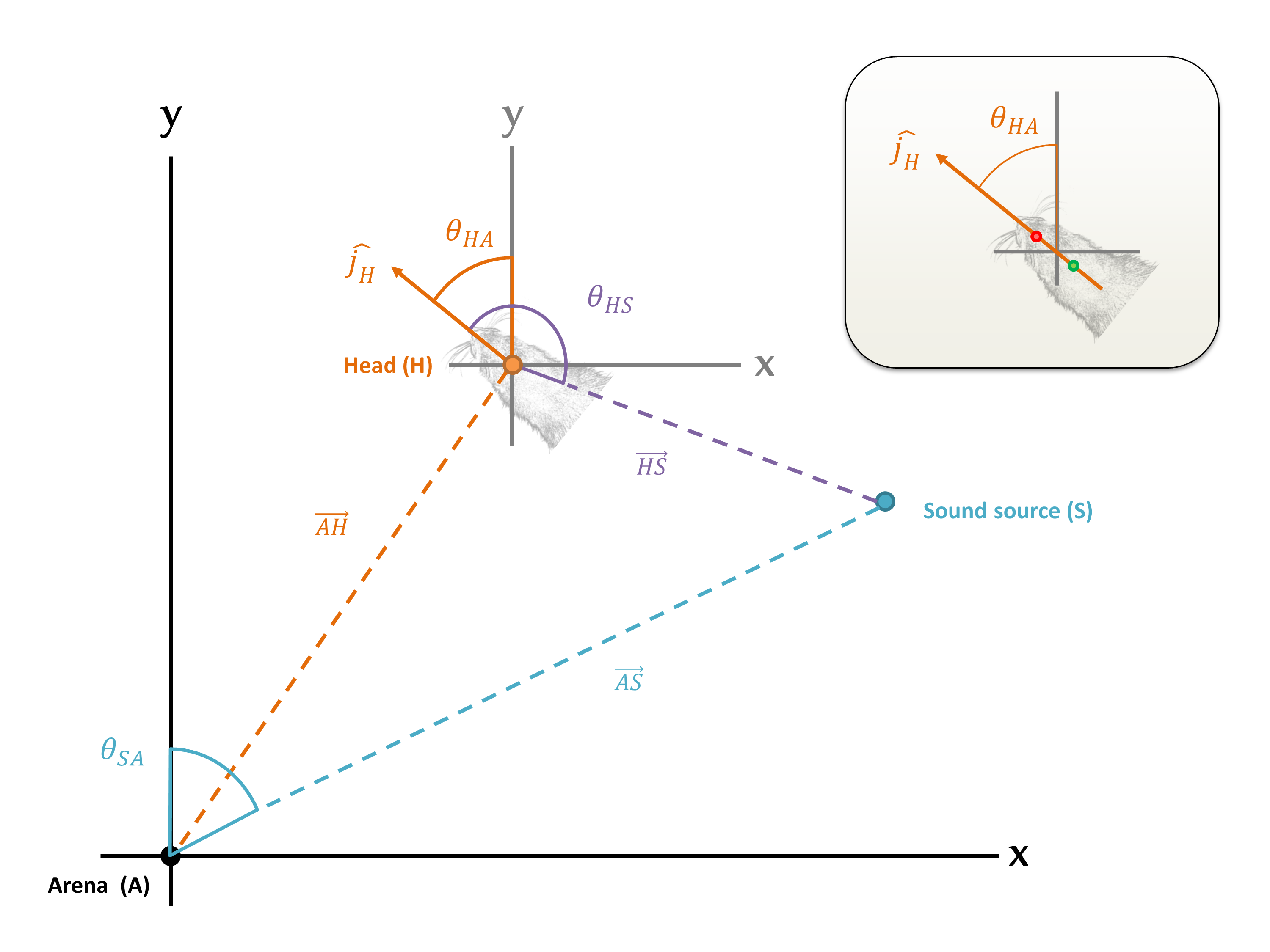

Supplement: S11 Fig — (TIF) [file pbio.2001878.s011.tif]
